# Snus: a compelling harm reduction alternative to cigarettes

**DOI:** 10.1186/s12954-019-0335-1

**Published:** 2019-11-27

**Authors:** Elizabeth Clarke, Keith Thompson, Sarah Weaver, Joseph Thompson, Grant O’Connell

**Affiliations:** 1 Imperial Brands Plc, 121 Winterstoke Road, Bristol, BS3 2LL UK; 2 Elucid8 Holdings Ltd, Ballymena Business Centre, 62 Fenaghy Road, Ballymena, BT42 1FL UK

**Keywords:** Snus, Harm reduction, Public health, Epidemiology, Cigarette smoking, Tobacco, Health effects

## Abstract

Snus is an oral smokeless tobacco product which is usually placed behind the upper lip, either in a loose form or in portioned sachets, and is primarily used in Sweden and Norway. The purpose of this review is to examine the reported effects of snus use in relation to specified health effects, namely lung cancer, cardiovascular disease, pancreatic cancer, diabetes, oral cancer and non-neoplastic oral disease. The review also examines the harm reduction potential of snus as an alternative to cigarettes by comparing the prevalence of snus use and cigarette smoking, and the reported incidence of tobacco-related diseases across European Union countries. The scientific literature generally indicates that the use of snus is not a significant risk factor for developing lung cancer, cardiovascular disease, pancreatic cancer or oral cancer. Studies investigating snus use and diabetes have reported that high consumption of snus (estimated as being four or more cans per week) may be associated with a higher risk of developing diabetes or components of metabolic syndrome; however, overall results are not conclusive. Snus use is associated with the presence of non-neoplastic oral mucosal lesions which are reported to heal rapidly once use has stopped. The most recent Eurobarometer data from 2017 reported that Sweden had the lowest prevalence of daily cigarette use in the European Union at 5% whilst daily “oral tobacco” use was reported to be 20%. European data published by the World Health Organisation in 2018 indicated that Sweden had the lowest rate of tobacco-related mortality and the lowest incidence of male lung cancer. Overall, prevalence statistics and epidemiological data indicate that the use of snus confers a significant harm reduction benefit which is reflected in the comparatively low levels of tobacco-related disease in Sweden when compared with the rest of Europe. The available scientific data, including long-term population studies conducted by independent bodies, demonstrates that the health risks associated with snus are considerably lower than those associated with cigarette smoking.

## Introduction

Snus is a moist oral tobacco product which is placed behind the upper lip, either loose or in portioned sachets, which resemble miniature tea bags. Air-cured tobacco is ground, mixed with salt and water and then processed under strict quality and regulatory controls using a technique similar to pasteurisation. Snus is distinctly different to other oral tobacco products due to the unique manufacturing process involved [1].

Within the European Union (EU), the sale of snus is prohibited by legislation in all countries except Sweden which has an exemption [2]. Snus is also available in Norway as it is not an EU member country and, as such, is not bound by EU legislation [1]. Swedish Match (a snus manufacturer) recently challenged the validity of the ban for a second time, arguing that new scientific data has shown it to be less harmful than cigarettes. However, after reviewing the evidence in November 2018, the European Court of Justice ruled to uphold the ban on snus [3].

The purpose of this review is to examine the effects of snus use in relation to certain health endpoints namely lung cancer, cardiovascular disease, pancreatic cancer, diabetes, oral cancer and non-neoplastic oral disease. The studies discussed in this review refer specifically to the use of snus in European populations only. This is because smokeless oral tobacco products from other geographical regions are manufactured under different conditions, and frequently contain substances other than tobacco such as slaked lime and areca nut. Such products are therefore regarded as being substantially different from snus [4]. The review also examines the harm reduction potential of snus as an alternative to cigarettes by comparing the prevalence of snus and cigarette use, and the reported incidence of tobacco-related diseases across EU countries.

## Prevalence data

**Fig. 1:**
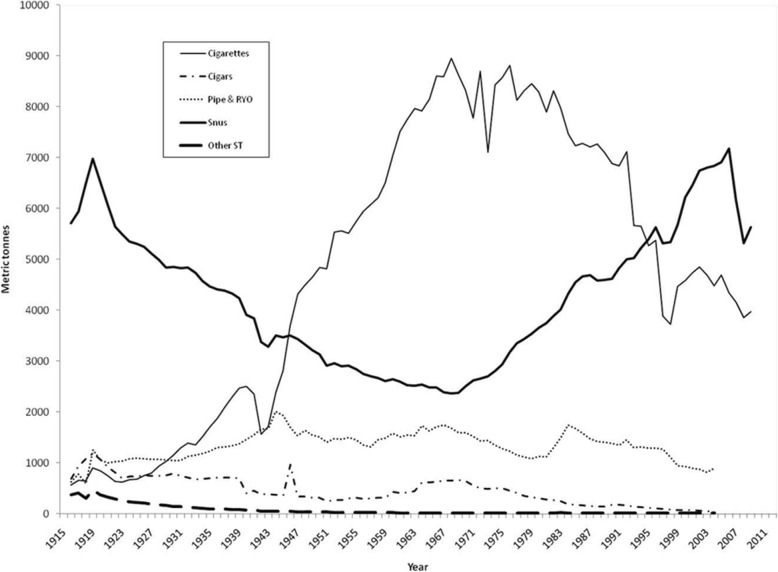
Tobacco sales in Sweden according to product category, 1916–2008. The data refers to deliveries made to points of sale, adjusted for recalls and returns. Note that official statistics cannot adjust for tax-free or cross-border trading or illegal entries into or out of the country. RYO, roll-your-own; ST, smokeless tobacco. Taken with permission from [5]

Sweden has the longest history of snus use in Europe. Snus was reportedly introduced into Sweden in 1637 and became popular among aristocratic men and women. Snus use reached record levels in 1919 but started to decline with the introduction of cigarettes (Fig. 1; taken from [5]). Figure 1 shows that generally, from the 1970s to 2005, the prevalence of smoking declined in Sweden whilst snus use increased in popularity.

**Fig. 2:**
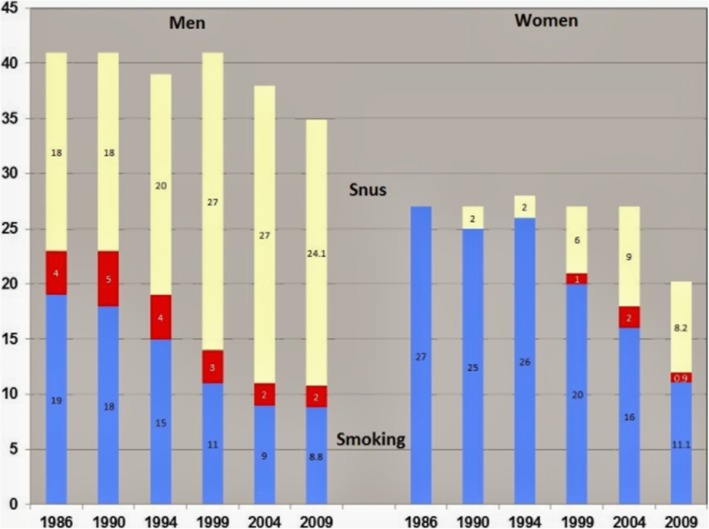
Percentage prevalence of snus use (yellow), cigarette use (blue) and dual use (red) among men and women aged 25 to 64 in Northern Sweden from 1986 to 2009. Taken with permission from [7]

Snus has now been used widely by consumers in Sweden over the past four to five decades which is sufficient time for epidemiological studies to assess potential effects of its use on health. In 2016, The UK Royal College of Physicians stated that the trends in smoking and snus use indicated that snus had become a substitute for smoking particularly among men [6]. This is corroborated by data from 1986 to 2009 showing that the prevalence of snus use has increased over time, above and beyond that of cigarettes, especially in men (Fig. 2; taken from [7]).

Swedish national statistics indicate that snus has been more popular than smoking among men since around 1996 [1]. Among women, daily snus use is much lower. However, in recent years this has increased from 1% in 1996 to 4% in 2015. Recently, the growth of daily snus use among men has slowed; rates were 19% in both 1996 and 2015 [8], and the most recent Eurobarometer data from 2017 reported daily oral tobacco use at 20% [9]. The Swedish pattern of increasing snus use and declining cigarette smoking has also been observed in Norway.

**Fig. 3:**
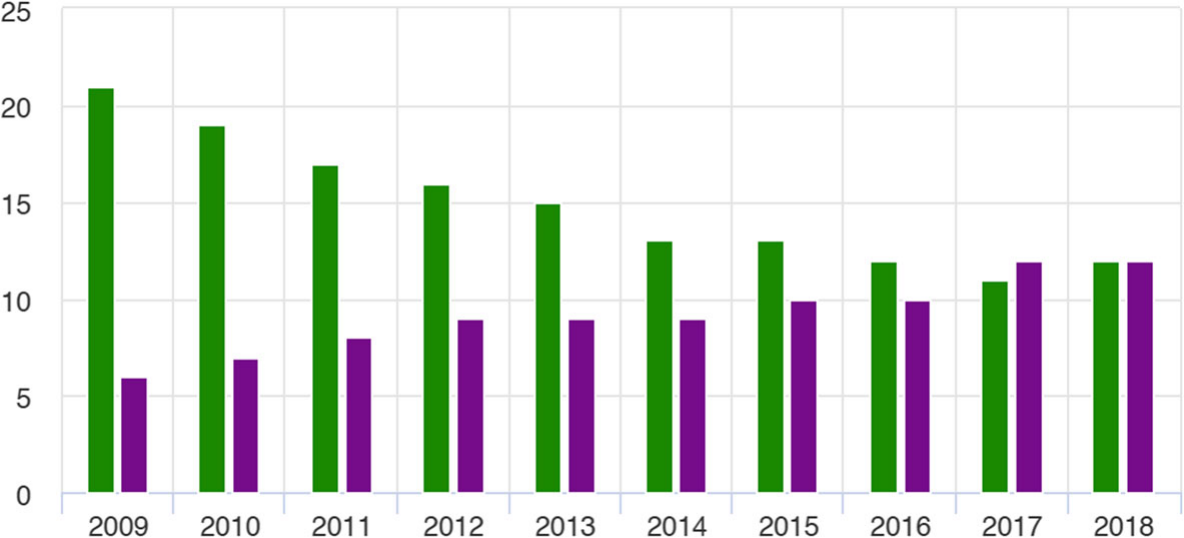
Percentage of the Norwegian population aged 16 to 14 who use snus or cigarettes on a daily basis stratified by product type (snus, purple; cigarettes, green) and year (2009 to 2018). Taken from [10]

According to Statistics Norway, smoking rates in Norway have declined over the past decade whilst snus use has increased over the same time period (Fig. 3; taken from [10]).

**Fig. 4:**
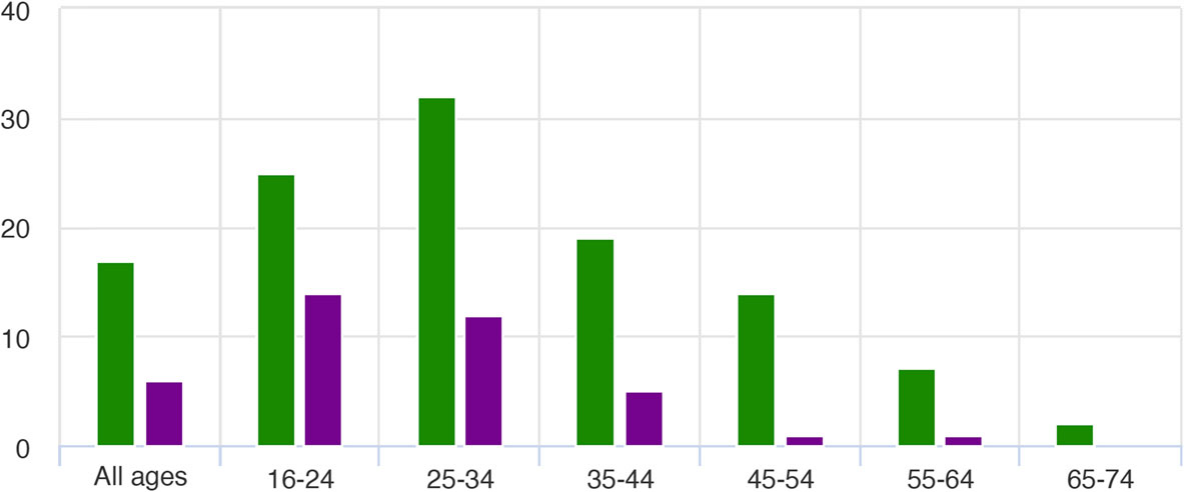
Percentage of the Norwegian population in 2017 who used snus on a daily basis stratified by gender (male, green; female, purple) and age. Taken from [11]

In 2017, 11% of the Norwegian population aged 16 to 74 were daily cigarette smokers, whilst 12% used snus daily. 2017 was reported as being the first year in which there were more daily snus users than cigarette smokers [11]. Statistics Norway reported that the prevalence of daily snus use is highest among young men below 35 years of age (Fig. 4; taken from [11]).

Norway shows a similar trend to Sweden with a higher proportion of males compared to females being daily snus users. The Norwegian data shows that snus is most popular among younger age groups (16- to 34-year-old age groups).

With respect to snus use within the EU, the *Special Eurobarometer 458* report examined the use of a range of tobacco products across the 28 EU member states in 2017 [9]. The Eurobarometer report specifically collected data regarding the use of “smokeless tobacco products” which included snus, chewing and nasal tobacco. The EU data indicated that smokeless tobacco products were used predominantly in Sweden with minimal regular use reported in a few other EU member states. This is expected given the EU regulatory framework as discussed previously.

**Fig. 5:**
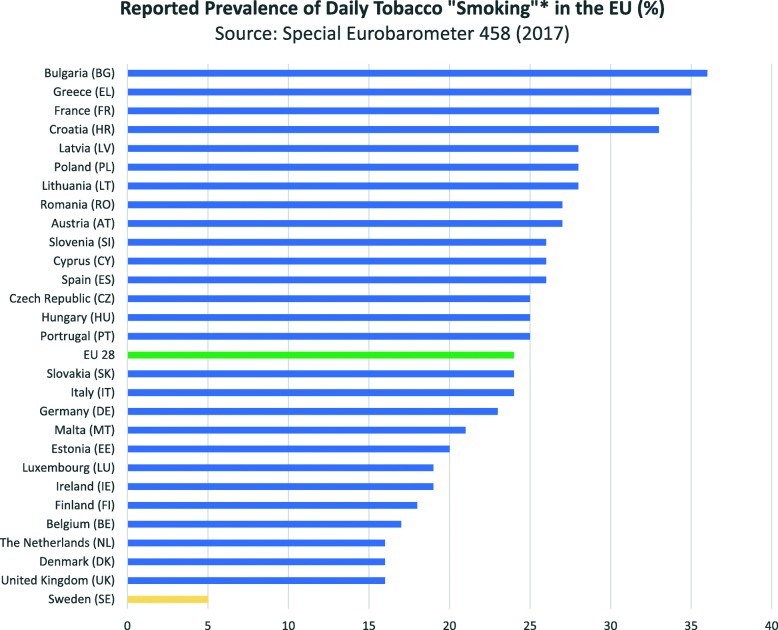
Percentage prevalence of daily tobacco “smoking” (*defined as the use of “boxed” cigarettes, hand-rolled cigarettes, cigarillos, cigars and pipe tobacco) across EU countries. Produced using data taken with permission from [9]. *The European Union does not endorse changes*, *if any*, *made to the original data and in general terms*, *to the original survey*, *and such changes are the sole responsibility of the author and not the EU*

Data collected from national surveys suggests that smoking prevalence is decreasing globally although trends vary substantially across countries and by gender [12, 13]. Smoking rates have declined substantially in Western and Northern Europe, notably in the UK and Nordic countries. In the UK, smoking rates dropped from over 80% in men in 1950 and approximately 40% in women in 1970, to approximately 20% in both sexes in 2012 [14]. Although smoking rates have also started to decrease in many other European countries, rates are higher in Eastern European and Southern European countries. According to the most recent Eurobarometer data, the overall proportion of smokers in the EU has remained stable since 2014 with just over a quarter of respondents reporting to be smokers. Over half of respondents report never having smoked (53%) whilst 20% of respondents claim to be former smokers [9]. Factory-made cigarettes were reported to be the most commonly used smoked tobacco product for each EU country [9]. Swedish respondents reported the lowest rate of daily smoking by a considerable margin (5%) compared to the UK which reported the second lowest figure (16%) (Fig. 5).

## Tobacco smoking epidemiology

Since the early 1950s, thousands of epidemiological, clinical and scientific publications have reported the adverse health consequences of smoking. Based on these findings, numerous public health bodies including the UK Royal College of Physicians, the US Surgeon General and the International Agency for Research on Cancer have concluded that smoking is causally associated with numerous diseases [6, 15, 16].

According to the European Parliament, tobacco consumption is reportedly responsible for nearly 700,000 deaths in the EU every year, with half of smokers dying an average of 14 years earlier than a never-smoker [17]. The World Health Organisation (WHO) European Region reports the highest proportion of deaths attributable to smoking compared to the rest of the world. The WHO has estimated that smoking is currently responsible for 16% of all deaths in adults over 30 years of age in Europe, which is above the global average of 12% [18]. Lung cancer has been found to be the most common cancer-related cause of death in Europe [19], and smoking has been associated with an increased risk of squamous cell carcinoma, adenocarcinoma, large cell carcinoma and small cell carcinoma of the lung [16]. Swedish males reportedly have the lowest rate of lung cancer and lowest rate of tobacco-related morality in Europe [20].

## Harm reduction potential of snus

**Fig. 6:**
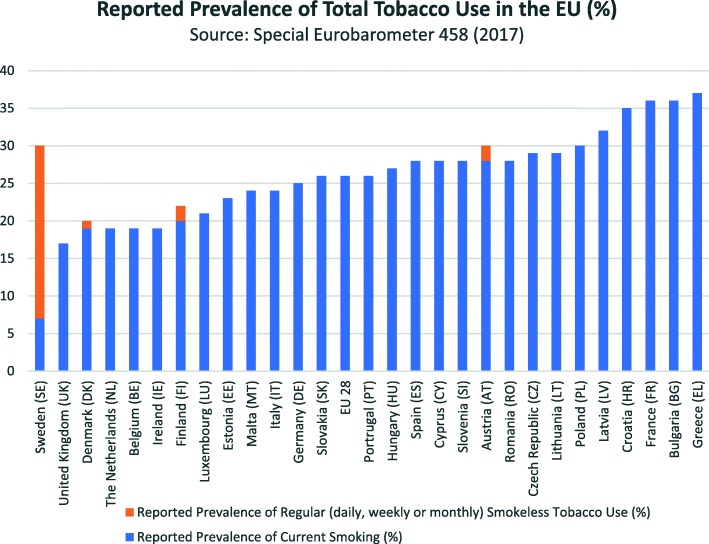
Percentage prevalence of cigarette and smokeless tobacco users stratified by country within the EU in 2017. Produced using data taken with permission from [9]. *The European Union does not endorse changes*, *if any*, *made to the original data and in general terms*, *to the original survey*, *and such changes are the sole responsibility of the author and not the EU*

Tobacco harm reduction is a strategy intended to reduce the health risks associated with smoking to individuals and the wider society. This may be achieved by using an alternative product which is less harmful than cigarettes. Figure 6 (produced using data taken from [9]) shows that total tobacco consumption in Sweden is within a similar range to other European countries. However, smoking-related mortality is markedly lower [20]. The comparably low incidence of smoking-related mortality in Swedish males [21] may be explained by snus being a viable, less harmful alternative to cigarettes.

Nutt et al. assessed the harm conferred by a range of different nicotine and tobacco products [22] according to a set of criteria including non-health-related measures. Snus was estimated to confer only 5% of the harm of cigarettes.

The epidemiology relating to the use of snus indicates it is substantially less harmful to health than smoking [23, 24]. In 2007, The UK Royal College of Physicians stated there are no clearly established causes of premature death associated with snus use [25]. Literature reviews have estimated that users of snus have at least 90–95% less smoking-related mortality, with minimal reduction in life expectancy, if any at all [26, 27]. The health benefits of smokers who completely transition to snus use are similar to those reported for smoking cessation [28].

The Scientific Committee on Emerging and Newly Identified Health Risks (SCENIHR) [23] concluded that snus use carried an overall risk reduction close to 100% for respiratory disease (lung cancer, chronic obstructive pulmonary disease and pneumonia), at least 50% for cardiovascular disease and at least 50% for oral and pharyngeal, oesophageal and pancreatic cancers compared to cigarette smoking. It should also be noted that the levels of several harmful compounds (such as tobacco-specific nitrosamines, lead and aflatoxins) in snus have decreased over the past two decades, primarily due to advances in production and processing techniques.

## Evidence for specific disease endpoints

The endpoints discussed specifically in this review are lung cancer, cardiovascular disease, pancreatic cancer, diabetes, oral cancer and non-neoplastic oral disease. The data are summarised in the corresponding supplementary tables (Additional files 1, 2, 3, 4, 5, 6, 7 and 8).

### Lung cancer

Respiratory diseases, predominantly lung cancer, chronic obstructive pulmonary disease (COPD) and pneumonia, are reported to account for 46% of all deaths due to smoking [23]. With snus use, there is negligible risk of lung cancer since there is no combustion and exposure to nicotine and tobacco constituents does not occur via inhalation through the respiratory tract [29].

**Fig. 7:**
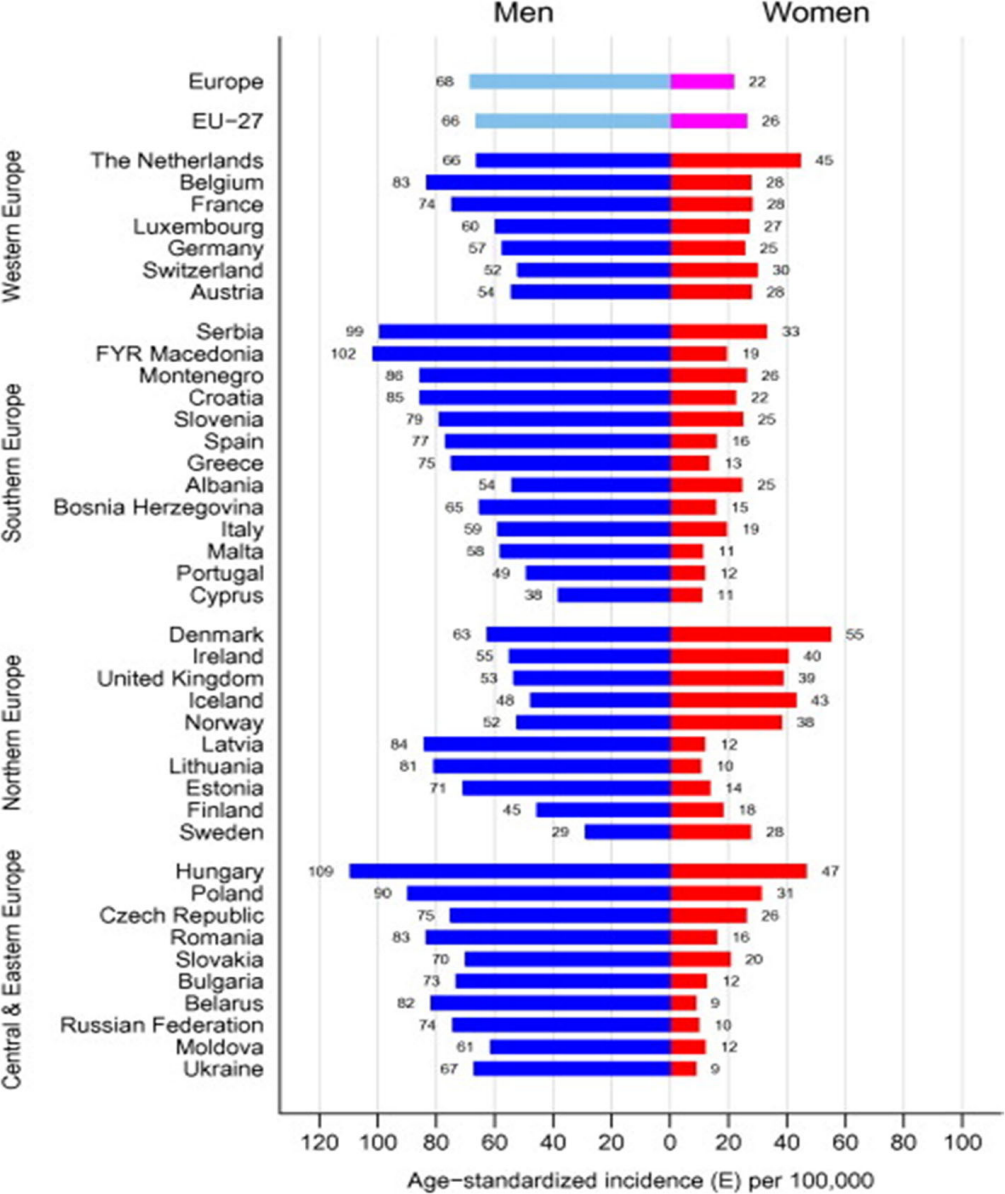
Age-standardised incidence rate of lung cancer stratified by gender, age and country in the EU in 2012. Taken with permission from [20]

Lung cancer is the leading cause of cancer death among men in all European countries except Sweden, see Fig. 7, taken from [20]. One study [30] estimated that if the Swedish male lung cancer mortality rate was extrapolated to the rest of the EU, there would be a 54% reduction in male mortality from lung cancer.

There are two epidemiological studies, that have reported the potential effects of snus use on lung cancer risk [31, 32]. One study [31] retrospectively analysed data from a large cohort of Swedish construction workers (*n* = 125,576) and reported an insignificant relative risk (RR) of 0.8 with 95% confidence interval (CI) of 0.5 to 1.3 for ever-users of snus, compared with never-users of any tobacco product. The second study [32] prospectively examined a cohort of Norwegian males (*n* = 10,136) and also reported no increased risk among ever-users of snus (RR 0.8, 95% CI 0.61–1.05).

### Cardiovascular disease

Cardiovascular disease is a broad term referring to a range of conditions that affect the heart and the blood vessels including ischaemic heart disease (also known as coronary heart disease) which can lead to myocardial infarction and stroke. Cardiovascular diseases are complex, chronic conditions and there are many different risk factors associated with their development such as being overweight (as indicated by an increased body mass index (BMI)), high blood pressure, high cholesterol, diabetes, being physically inactive, tobacco smoking, excessive alcohol consumption, family history of cardiovascular disease, ethnicity, gender and age [33]. The potential association between snus use and cardiovascular disease is based on the presence of nicotine [29], which is a mild stimulant.

### Ischaemic heart disease/myocardial infarction

Three epidemiological studies, all based on data from the Swedish construction workers cohort, have reported an increased risk of myocardial infarction associated with current snus use [34–36]. The first study [34] (*n* = 135,036) found that the age-adjusted RR of dying from cardiovascular disease was 1.4 (95% CI 1.2–1.6) for smokeless tobacco users and 1.9 (95% CI 1.7–2.2) for smokers of fifteen or more cigarettes per day compared with those who reported never using tobacco. Among men aged between 35 and 54 years at the start of follow-up, the RR was reportedly 2.1 (95% CI 1.5–2.9) for smokeless tobacco users and 3.2 (95% CI 2.6–3.9) for smokers (adjusted for BMI, blood pressure and history of heart symptoms). The authors of this study concluded that tobacco users face a higher risk of dying from cardiovascular disease compared to those who do not use tobacco, although the risk is lower for smokeless tobacco users. The second study [35] (*n* = 118,395) reported that the multivariable-adjusted RRs for myocardial infarction in ever-snus users were 0.91 (95% CI 0.81–1.02) for nonfatal cases and 1.28 (95% CI 1.06–1.55) for fatal cases. Heavy snus users (estimated as being use of more than 50 g of snus per day) were reported to have a RR of fatal myocardial infarction of 1.96 (95% CI 1.08–3.58). The third study [36] examined data from two cohorts; the Swedish construction workers cohort (*n* = 118,425) and the Uppsala Longitudinal study of adult men (*n* = 1,076). In the former study, the authors reported adjusted hazard ratios (HRs) of 1.28 (95% CI 1.00–1.64) for heart failure and 1.28 (95% CI 0.97–1.68) for non-ischaemic heart failure for current snus users compared to those who had never used tobacco. In the latter study, the adjusted HR associated with heart failure for current snus use was reported to be 2.08 (95% CI 1.03–4.22) compared to non-use. It should be noted that none of these studies [34–36] took into account the smoking history of snus users which is a major confounding factor. In addition, the studies did not consider alcohol consumption, medical history or any changes regarding tobacco product use. All data were self-reported, and the studies used only male participants.

A case-control study [37] (*n* = 585 cases, *n* = 589 controls) which specifically examined the risk of myocardial infarction in smokers, snus users and non-tobacco users, reported an age-adjusted odds ratio (OR) for myocardial infarction of 0.89 (95% CI 0.62–1.29) for snus users and 1.87 (95% CI 1.40–2.48) for cigarette smokers compared with non-tobacco users. The same research group confirmed their findings in a further case-control study [38] (*n* = 687 cases, *n* = 687 controls) which reported that after adjustment for multiple cardiovascular factors, the OR for myocardial infarction was 3.53 (95% CI 2.48–5.03) in smokers and 0.58 (95% CI 0.35–0.94) for snus users. The authors of this study concluded that the risk of myocardial infarction was not increased in snus users and that nicotine is unlikely to be an important contributor to ischemic heart disease in smokers.

Other epidemiological studies [39–44] have reported no association between current snus use and increased risk of myocardial infarction or excess risk of ischaemic heart disease. A study which used data from the Swedish construction workers cohort to investigate the incidence of atrial fibrillation found that snus use is unlikely to confer any important increase in risk [45].

SCENIHR have stated that smokeless tobacco products appear to increase the risk of death *after* myocardial infarction, but that its use is not associated with an increased risk of myocardial infarction [23]. However, this finding is based on smokeless tobacco studies from the US and South East Asia and does not exclusively consider Swedish snus.

### Stroke

There are six Swedish epidemiological studies [29, 34, 40, 42, 43, 46, 47] and one meta-analysis [48] published to date which have specifically investigated the potential adverse effect of snus use on the incidence of stroke. The earliest study [34] was based on data from the male Swedish construction workers cohort (*n* = 135,036) and reported a statistically insignificant age-adjusted RR of 1.9 (95% CI 0.6–5.7) for snus users (aged 35 to 54 years at entry into the study) compared to non-tobacco users. Data were reportedly adjusted for BMI, blood pressure and unspecified “history of heart symptoms”. However, the analysis was based on only four cases of stroke among the smokeless tobacco users in the cohort. The study did not adjust for smoking history or alcohol consumption.

Four other studies all reported insignificant RRs for current snus use and stroke [29, 40, 42, 43]. One study [46] (*n* = 118,465) which investigated morbidity and mortality from stroke and its subtypes (ischaemic, haemorrhagic and unspecified stroke) reported no overall association with snus use. However, an increased RR was reported specifically for fatal ischaemic stroke and current snus use (1.72, 95% CI 1.06–2.78).

These six studies were subsequently included in a meta-analysis [48] which reported no association of stroke with current snus use, with a combined RR estimate of 1.05 (95% CI 0.95–1.15) for the whole population and 1.06 (95% CI 0.96–1.17) for never smokers.

The most recent publication to date [47] investigated the incidence of, and survival after stroke, both overall and according to subtypes. The study conducted a pooled analysis of eight Swedish prospective cohort studies (*n* = 130,485). No associations were observed between the use of snus and the overall risk of stroke (HR 1.04, 95% CI 0.92–1.17) or for any specific stroke subtype. However, the authors of this study reported an OR of 1.42 (95% CI 0.99–2.01) for “28-day case fatality” among users of snus who had experienced a stroke, and the HR of death during the follow-up period was reported to be 1.32 (95% CI 1.08–1.61). However, the authors acknowledge they could not differentiate whether the reported associations were due to snus use or social disadvantage.

A number of the above studies either reported on or referred to an increased risk of stroke associated with smoking. On the basis of the results reported for snus, several authors [29, 40, 44] suggested that nicotine is unlikely to contribute significantly to the pathophysiology of stroke.

## Other cardiovascular risk factors

Several studies have investigated other aspects of cardiovascular disease including hypertension, atherosclerosis and markers for metabolic syndrome (such as serum triglycerides). The following relevant studies were all based in Sweden.

Two small crossover studies [49, 50] (*n* = 9 and *n* = 135, respectively) and a cross-sectional study [51] (*n* = 20) examined the acute effects of snus on blood pressure and all reported significant increases (*p* < 0.05) at rest. This was attributed to the mild stimulating effects of nicotine, as similar (or higher) increases in blood pressure were seen in smokers [50, 51]. Two other studies, both based on data from the Swedish construction workers cohort (*n* = 97,586 and *n* = 120,930, respectively) also reported a significant effect of snus use on blood pressure [52, 53]. Hergens et al. [53] reported that snus users who had never smoked and had normal blood pressure at baseline had a significantly higher risk of developing hypertension at follow-up 15 years later (OR 1.36, 95% CI 1.07–1.72), after adjustments for age and BMI. The results could not be adjusted for diet, physical activity or education due to a lack of data; however, the authors note that the results for former snus users were less consistent and not as statistically significant as those for current snus use, indicating that the effects on blood pressure may be attenuated to some degree with cessation of snus use.

A small study [54] (*n* = 30) investigating systolic and diastolic heart parameters before and after snus use in a group of healthy volunteers reported significantly altered pulse and blood pressure responses (*p* < 0.01). Tests showed a significant decrease in ventricular relaxation and reduced diastolic heart function in the left and right ventricles. The authors state that although the mechanism behind these observations is likely to be complex, nicotine pharmacology is likely to be the main factor.

A further study [55] reported that the resting heart rate of habitual snus users (*n* = 24) who quit for 6 weeks was significantly lower (decreased by 5 ± 7 beats per minute) compared to controls (*n* = 11) who maintained snus use. However, differences in blood pressure were not significantly different.

A number of other studies found no significant increase in blood pressure among current snus users compared with those who did not use snus [42, 56–60]. A review of the epidemiological evidence relating to snus and blood pressure concluded that the overall evidence does not demonstrate that snus use is linked to developing hypertension [48].

Current and former snus use was associated with higher serum triglycerides compared to never-users of tobacco in a study [59] examining tobacco product use and cardiovascular risk factors, in a sample of males (*n* = 391) who were 58 years of age. However, former snus users reportedly had higher levels than current snus users; it should be noted that former snus users had the highest number of “cigarette years”. There were no associations of snus use with other measures of sub-clinical atherosclerosis. However, several associations were reported for tobacco smokers.

A large, longitudinal study [61] (*n* = 24,230) which investigated associations between several lifestyle factors, including snus use, and metabolic syndrome found that after a 10-year follow-up, high-dose consumption of snus (defined by the authors as more than four cans per week) at baseline was associated with an increased risk of some components of metabolic syndrome including raised triglycerides (OR 1.6, 95% CI 1.30–1.95) and obesity (OR 1.7, 95% CI 1.36–2.18), but not others, including hypertension and low-density lipoprotein cholesterol. A subsequent longitudinal study [62] (*n* = 880) which investigated snus use and the prevalence of metabolic syndrome and its components concluded no association between snus use and metabolic syndrome.

### Pancreatic cancer

There are several risk factors that may increase an individual’s risk of developing pancreatic cancer including age, smoking, being overweight, family history, pancreatitis and diabetes, according to Pancreatic Cancer UK [63].

To date, the largest study [64] investigating a potential link between snus use and pancreatic cancer used pooled data from the Swedish Collaboration on Health Effects of Snus Use. A total of 424,152 males were followed up for risk of pancreatic cancer through linkage to health registers with a reported total of 9,276,054 person-years of observation. Current snus use was not found to be associated with an increased risk of pancreatic cancer (HR 0.96, 95% CI 0.83–1.11) when compared to never-users of any tobacco product.

Two earlier epidemiological studies [31, 32] reported an association between snus use and an increased risk of pancreatic cancer. One study [31] retrospectively analysed data from a large cohort of Swedish construction workers (*n* = 125,576) and although no association was reported for the whole population (RR 0.9, 0.7–1.2), a RR of 2.0 (95% CI 1.2-3.3) was reported for never-smoking, ever-users of snus, compared with never-users of any tobacco product. The second study [32] prospectively examined a cohort of Norwegian males (*n* = 10,136) and reported that ever-use of snus was associated with a RR of 1.67 (95% CI 1.12–2.50) for pancreatic cancer; however, no association was found when the sample population was stratified for never smokers (RR 0.85, 0.24–3.07). Neither study controlled for important variables including alcohol consumption and diabetes. Boffetta et al. [32] used pooled data from a sample of the Norwegian population identified by the 1960 census and relatives of Norwegian migrants to the USA. As such, some participants may have used American smokeless tobacco products rather than Swedish snus, which may not be equivalent as discussed previously, and there may be additional sources of confounding including diet and socioeconomic status. The limitations of both studies are discussed at length in reviews of the scientific literature [65, 66].

### Diabetes

There are eleven primary research publications [56, 58, 61, 62, 67–73], all based in Sweden, which have investigated the potential effects of snus use on type II diabetes or related components of metabolic syndrome (such as glucose tolerance or insulin sensitivity).

Seven studies specifically examined the potential effect of snus use on type II diabetes [67–73]. The earliest study published in 2000 [67] suggested that snus use was associated with type II diabetes. The study used a cross-sectional design with over three thousand male participants, of whom just over half reported a family history of type II diabetes. The authors reported that current exclusive snus use was associated with a RR of 3.9 for developing type II diabetes (95% CI 1.1–14.3) compared with those who reported never using tobacco. The authors also reported a RR of 2.7 (95% CI 1.3–5.5) in current heavy snus users (defined by the authors as more than three cans per week). The findings were adjusted for age, BMI, family history of diabetes and alcohol consumption but did not consider smoking history among snus users.

Since the first publication in 2000, several subsequent studies have concluded that the risk of type II diabetes is not significantly increased by snus use [68–71]. A further study [72] which examined the risk of type II diabetes in a large group of middle-aged, male snus users (*n* = 2,383) who had never smoked, found no significant effects within the whole group. However, the risk of diabetes was reported to increase with higher weekly snus consumption. The authors found an OR of 2.1 (95% CI 0.9–4.9) with consumption of more than four cans of snus per week and an OR of 3.3 (95% CI 1.4–8.1) with consumption of more than five cans per week. The results were adjusted for age, BMI, glucose tolerance at baseline, physical activity and alcohol consumption. The authors reported a similar association in smokers who smoked more than fifteen cigarettes per day.

A small study [56] primarily investigating cardiovascular risk factors, reported that habitual snus users (*n* = 21) and tobacco smokers (*n* = 19) exhibited higher serum insulin levels compared with controls (*n* = 18), at similar blood glucose concentrations. However, the same authors later reported no association between snus use and glucose tolerance and insulin levels in a much larger study (*n* = 1,266) [58].

A large longitudinal study [61] (*n* = 24,230) investigated the association between snus use and the development of metabolic syndrome after a 10-year follow-up period. A high consumption of snus (more than four cans per week) at baseline, was associated with an increased risk of some components of metabolic syndrome, including raised triglycerides (OR 1.6, 95% CI 1.30–1.95) and obesity (OR 1.7, 95% CI 1.36–2.18). However, other components including hypertension, dysglycemia and low-density lipoprotein cholesterol were not increased.

A study [62] which enrolled 16 year olds (*n* = 880) and concluded after a 27-year follow-up period when participants were aged 43, reported that exclusive snus use was associated with several components of metabolic syndrome (such as raised triglycerides and high blood pressure) in a crude analysis, but that no association was observed with the more statistically robust multivariate models. The authors of this study concluded that snus use from adolescence through to mid-adulthood did not appear to increase the risk of metabolic syndrome; however, more research was required regarding the effects of dual use of snus and cigarettes.

A pooled analysis using data from five cohorts [73] (*n* = 54,531) reported no significant association of snus use and type II diabetes for current snus users compared to never-users. However, individuals who reported consuming between five and six cans per week had a HR of 1.42 (95% CI 1.07–1.87) and those who reported consuming seven or more cans per week had a HR of 1.68 (95% CI 1.17–2.41).

A meta-analysis [74] including each of the above publications (and several others deemed not relevant to this review, either because they were not based in Europe or were confounded by smoking), reported insignificant RRs for never-smoking current, former and ever-snus users and type II diabetes. In addition, no significant association was reported for related endpoints including impaired glucose tolerance. However, when high snus consumption was considered separately, a RR of 1.65 (95% CI 1.25–2.18) for type II diabetes was reported. The authors of the meta-analysis concluded that existing studies were somewhat limited and that further research would be required to confirm any relationship between snus use and the development of type II diabetes.

### Oral cancer

Mouth cancer, also known as oral cancer, is classified as such when a tumour develops in the lining of the mouth. It may occur on the surface of the tongue, the insides of the cheeks, the roof of the mouth (palate) or the lips or gums [75].

Of eight publications [31, 32, 76–80], five large, well-controlled studies, that adjusted for smoking and alcohol consumption reported insignificant RR values for the association of oral cancer with snus use [31, 32, 76–78]. One Danish study [79] (*n* = 644) reported tobacco use as a significant risk factor for developing oral cancer; however, the study did not stratify results by the type of tobacco used (i.e. smoked cigarettes, chewing tobacco and snus (described by the authors as snuff)). The authors reported that none of the participants were current snus users and that very few individuals within their sample had previously used snus.

The seventh study, conducted by Roosaar et al. [80], analysed a subset of data from the Swedish construction worker study (*n* = 9,976). The authors reported a statistically significant RR of 3.1 (95% CI 1.5–6.6) for combined oral and pharyngeal cancer among ever-daily users of snus compared to never-daily users. It should be noted that the reported RR was based on 11 cases of oropharyngeal cancer. When the analysis was further restricted to those who had never smoked, the authors reported a RR of 2.3 (95% CI 0.7–8.3). The refined RR was based on only five cases of oropharyngeal cancer and was not statistically significant for combined oral and pharyngeal cancer among daily users of smokeless tobacco compared to never-daily users. The authors reportedly adjusted the results for alcohol consumption and smoking. An observational study by Hirsch et al. [81] reported 16 cases of patients who developed oral cancer at the exact anatomical site where snus was placed daily. The authors concluded a potential link between the use of snus and oral cancer; however, several patients had a history of smoking, and alcohol consumption was not recorded. The authors did not collect any control data regarding the location of oral cancer in other snus users; therefore, it is difficult to contextualise the findings.

In 2016, Swedish Match submitted a modified risk tobacco product (MRTP) application to the FDA, which sought permission to remove a number of product health warnings; one of which related to oral cancer. The FDA rejected the MRTP application [82], citing the results published by Roosaar et al. [80] stating (regarding the literature relating to snus and oral cancer) that “although the few epidemiological studies conducted on snus products are inconsistent, the most recently published study found a three-fold increase in the risk of oral cancer associated with the use of snus products”.

A meta-analysis of the scientific studies investigating oropharyngeal and oesophageal cancer and snus use [48] found no association between the two. The Institute of Medicine in their assessment of the scientific basis for tobacco harm reduction [83] concluded that *some* smokeless tobacco products increase the risk of oral cavity cancer and that a dose-response relationship exists. However, the overall risk is lower than for cigarette smoking, and some products, such as snus, may have no increased risk at all.

### Non-neoplastic oral disease

Non-neoplastic oral disease includes oral mucosal lesions (including leukoplakia), periodontal and gingival diseases, tooth loss and dental caries.

### Oral mucosal lesions

Oral mucosal lesions may generally be defined as any abnormal change or swelling on the epithelial lining of the mouth, lips or gums, which do not contain any malignant or pre-malignant cells [84]. The most recent publication investigating the potential association between snus use and oral mucosal lesions [85] is a review article which has analysed all the relevant studies to date. There have been no subsequent experimental or epidemiological studies. The review article included 15 Scandinavian studies of which 14 examined the prevalence of oral mucosal lesions and one examined oral leukoplakia, a type of pre-malignant lesion [86].

Based on reported results, the authors of the review concluded that the use of snus markedly increases the risk of developing oral mucosal lesions. They note that the results were not significantly altered by adjustment for variables including age, cigarette smoking, alcohol consumption and dental hygiene. Oral mucosal lesions were generally reported to disappear with cessation of snus use.

### Periodontal and gingival diseases

Gum disease is a very common condition where the gums become swollen, sore or infected. The early state of gum disease is known as gingivitis; however, if left untreated, this may progress to periodontal disease which is more serious and can adversely affect the jawbone and tissues that support the teeth [87]. There are eight relevant Swedish publications to date [88–95] which have investigated the use of snus and various endpoints relating to gum disease.

One study [88] reported that snus users had a significantly increased gingival index, a measure of gum inflammation. However, others [89–92] reported no association with gum infection, inflammation or bleeding. Gum recession was considered by five of the seven publications [89, 90, 92–94]. One study [92] (*n* = 103) reported a significant increase in receding gums among adolescent snus users (OR 3.72, 95% CI 1.40–9.99) compared to controls, and a second study [93] (*n* = 252) reported that receding gums were observed in 23.5% of subjects who used loose snus but only 2.9% in those who used snus pouches. Three publications [89, 90, 94] reported no significant association between receding gums and snus use.

The largest publication [95] comprised of three epidemiological, cross-sectional studies (*n* = 1,625), examined the potentially adverse effects of current smoking and the use of snus on periodontal health compared with non-tobacco users. The authors reported that cigarette smokers had a significantly higher risk of severe periodontitis compared with non-tobacco users and users of snus; the authors stated that using snus did not seem to be a risk factor for periodontitis. None of the other studies cited in this section reported a link between snus use and the presence of periodontal disease.

### Tooth loss and dental caries

One study [96], based on a sample of 14 to 19-year olds (*n* = 2,145), reported that the mean number of decayed, missing and filled teeth was significantly higher (*p* < 0.001) among snus users compared to non-users. It should be noted that the authors of this study did not adjust for age and that snus users were generally older than the non-users. Rolandsson et al. [90] (*n* = 80) reported no significant association between the use of snus and the number of fillings and the number of teeth present. Bergström et al. [91] also concluded that snus use was not related to the number of teeth present. The largest study [97] (*n* = 1,625) based on three epidemiological, cross-sectional studies over 20 years reported that “daily smoking and use of snus does not increase the risk of dental caries”. Hellqvist et al. [98] (*n* = 102) reported that there was no statistically significant difference in the prevalence of dental caries between habitual snus users (those individuals with over 10 years of use) and non-tobacco users.

A review of the experimental and epidemiological studies published in Scandinavia and the USA between 1963 and 2007 relating to the above oral endpoints [85] reported a strong association between current use of smokeless tobacco, particularly snus, with the prevalence of oral mucosal lesions. The authors stated the current body of scientific literature provided “suggestive” evidence of an association of snus use with receding gums. However, the authors noted that interpretation for other endpoints was limited by study weaknesses including poor confounding control.

## Dual use, gateway and cessation

The potential effects of snus use when combined with cigarette use (dual use), the potential role of snus use to act as a gateway to cigarette use and the role of snus to aid smoking cessation will be discussed in this section of the review.

### Dual use

There is concern that uptake of secondary snus use among daily smokers may result in permanent dual use and increase the risk of tobacco-related morbidity and mortality above the risk associated with single-product use [99].

A review of eleven Swedish publications investigating the prevalence of dual use [48] reported average percentages as low, rarely exceeding 10%. The low percentages indicated by these studies are consistent with the Swedish prevalence data as presented in Fig. 2. The level of current dual use is similarly low in Norway [100]. The prevalence of dual use is higher among Swedish adolescents compared to adults [101, 102] and the reported frequency of ever dual use is much higher than the frequency of current dual use [103]. After analysing the data relating to dual use, it was suggested [104] that the observed trends may be explained by adolescents trying both products, and after a period of dual use, choosing one product over the other.

Another review [28] reported that more than eight out of ten smokers who started using snus had quit daily smoking and that almost one third no longer used any form of tobacco on a daily basis. The authors concluded that dual use appeared to represent a transient rather than permanent state and that uptake of snus use among smokers may be a stepping stone towards changing or quitting their tobacco use.

The health risks associated with dual use have not been widely studied. However, one review [105] considered studies where health risks could be compared in dual users, those who report exclusively using snus or cigarettes, and never-users of either product. Of 51 interactions analysed in the review, only one study for hypertension in pregnancy reported a significantly (*p* < 0.05) higher risk for dual product use than for cigarette smoking alone (RR 2.72, 95% CI 1.30–5.69). It should be noted that the results of this study were based on only seven cases of hypertension in pregnancy; it is possible the results may be confounded by the development of gestational hypertension, and product use was self-reported.

In his review [104], Lee concluded that the reduced health risks associated with dual use were likely to be due to a lower number of cigarettes smoked per day. Cigarette consumption tends to be lower in dual users compared to those who only smoke cigarettes.

### Gateway

The gateway hypothesis posits that, among persons who have not previously smoked, users of snus are more likely than non-users to take up smoking. A review of the evidence [106] which examined gateway effects in Sweden suggested that snus appeared to lead users away from smoking rather than towards it and is an important reason why Sweden has the lowest rates of tobacco-related disease in Europe.

Since 2006, several studies have investigated potential gateway effects. One prospective study [107] (*n* = 2,938) reported that adolescents who initiated tobacco use with cigarettes had a non-significantly increased adjusted OR of progressing to smoking when compared with snus starters (OR 1.42; 95% CI 0.98–2.10). The authors concluded that the proportion of adolescent smoking attributable to a potential induction effect of snus is likely to be small.

A longitudinal cohort study of Swedish adolescents [108] (*n* = 649) examined predicting factors for smoking in late adolescence. A multivariable analysis found that female gender (OR 1.64, 95% CI 1.08–2.49), medium and low self-esteem (medium: OR 1.57, 95% CI 1.03–2.38, low: 2.79, 95% CI 1.46–5.33), a “less negative attitude” towards smoking (OR 2.81, 95% CI 1.70–4.66) and ever using snus (OR 3.43, 95% CI 1.78–6.62) were significant factors. However, there were reportedly very few snus users and smokers at baseline, and a high dropout rate of snus users at follow-up, which adds uncertainty to the findings.

A 15-month longitudinal Swiss study [109] aimed to test whether snus and nasal snuff (a form of smokeless tobacco inhaled directly into the nasal cavity) use decreased smoking incidence and prevalence in a large sample of young men (*n* = 5,198). Snus and nasal snuff were not observed to confer any benefits regarding cigarette smoking initiation, cessation or reduction among participants. The authors noted that snus was not legally available in Switzerland and that smokeless tobacco is not highly promoted and does not benefit from tax incentives, as was the case during the 1970s in Sweden.

Regarding snus and the gateway hypothesis, SCENIHR [23] state that Swedish data do not support the hypothesis that such products are a gateway to smoking. The UK Royal College of Physicians [6] state that the observed smoking and snus trends in Sweden indicate that snus has become a substitute for smoking, particularly among men.

One review [28] of supposed gateway effects and the smoking and snus prevalence trends in Sweden challenged the credibility of the gateway hypothesis. The authors state that if any gateway effect did exist, it is dwarfed by other factors which are net protective, as the evidence strongly points towards snus playing a positive preventative role in smoking prevalence.

### Cessation

Seven studies [110–116] investigating the effect of snus on smoking cessation were identified in the scientific literature; four were conducted in Scandinavia [110–113] and three in non-Scandinavian countries [114–116].

One Scandinavian study [110] investigated twelve variables and their interactions as correlates of smoking cessation among regular smokers in the population-based Swedish Twin Registry (*n* = 14,715) and concluded that snus use was the strongest independent correlate of smoking cessation. Other correlates included nicotine dependence score, education and socioeconomic status. A study [111] that retrospectively examined the association between snus and smoking behaviour in males split into two age groups found six smoking quitters per smoking starter was attributable to snus in the younger age group. In the older cohort, there were slightly more than two quitters per starter. This study suggested that snus contributed to the reduction of smoking among Swedish males in the 1990s. A large Norwegian study [112] (*n* = 10,441) examined seven cross-sectional data sets collected between 2003 and 2008. The authors of this study reported that the quit ratio (quitters: ever-smokers) for smokers who used snus was significantly higher than for those with no experience of using snus in six of seven data sets. Pooled data suggested the primary reason for snus use among daily users was to quit smoking. The findings were consistent with Swedish data, supporting that snus may play a role in smoking cessation.

Rutgvist et al. conducted a survey [113] (*n* = 6,008) to evaluate the methods used by Swedish smokers to quit. The results confirmed and extended previous studies that found most smokers quit unassisted. In addition, snus was the most frequently reported cessation aid among male smokers, whereas usage of pharmaceutical nicotine was more prevalent among females. Use of snus at the latest quit attempt appeared to be associated with a significantly higher success rate among males.

With respect to countries outside of Scandinavia, a study conducted in the USA [114], which evaluated twelve methods used within a large cohort of males motivated to quit smoking (n > 4.3 million), reported that those who switched to snus had the highest rate of success (73%).

A 2-week study conducted in New Zealand [115] investigated the acceptability of snus, “Zonnic” (a non-tobacco, oral nicotine delivery product) and nicotine gum as smoking cessation aids. Participants (*n* = 63) reported a preference for snus and Zonnic and both were reportedly effective in reducing smoking and desire to smoke. The authors concluded that longer-term studies were warranted to test efficacy for long-term quit rates.

A Swedish Match sponsored trial [116] (*n* = 319) investigated the efficacy of snus as a smoking reduction and cessation aid in Serbia. At 24 weeks, a greater than 75% reduction in smoking was significantly (*p* < 0.01) more likely to be reported in the snus group compared to the placebo group. The results in this study were biologically verified.

A review [106] of smoking prevalence, snus use and associated effects on public health in Sweden suggested that the low rate of male smoking combined with high rate of snus use indicated the displacement of smoking by snus. The authors concluded that snus use prevents rather than promotes smoking and has contributed a net public health benefit in Sweden. However, the 2008 SCENIHR report [23] and the 2016 Cochrane review [117] concluded there was insufficient evidence to determine whether snus could aid long-term smoking cessation. The SCENIHR report was published prior to the seven studies cited above and was based on US research and the Cochrane review only included the Swedish Match sponsored trial when reviewing the effectiveness of different smoking interventions.

## Conclusion

This review found that the health risks associated with snus use, where nicotine is decoupled from harmful tobacco smoke, are considerably lower than those associated with smoking cigarettes. Further, snus appears to be a viable alternative to smoking tobacco, is acceptable to consumers and does not act as a gateway product to smoking cigarettes. Snus should therefore be regarded as a reduced risk product relative to cigarettes. These findings are in keeping with those reached recently by the UK Royal College of Physicians [25]. Snus as an alternative to cigarettes has the potential to deliver enormous harm reduction benefits as demonstrated in Sweden, particularly in reducing the incidence of lung cancer and cardiovascular disease of which smoking is a known cause, where the product can be marketed and sold to adult smokers widely. This review also shows that since the European Union implemented a ban on the sale and marketing of snus in 1992, a substantial and independent scientific evidence base has confirmed the harm reduction potential of snus. The EU ban on the marketing and sales of snus should be reviewed in line with this scientific evidence. If the ban on the sale of snus in the EU was lifted, snus could represent an opportunity to deliver extensive public health benefits across Europe as a strategy for harm reduction.

## Supplementary information

**Additional file 1: Table S1.** Epidemiological studies investigating the association between snus use and lung cancer. Those epidemiological findings which are statistically significant (either protective or causative) are highlighted in red. N/A; not applicable. Klimisch Score adapted from *Regulatory Toxicology and Pharmacology* (1997) **25**, 1-5 [118].

**Additional file 2: Table S2.** Epidemiological studies investigating the association between snus use and myocardial infarction. Those epidemiological findings which are statistically significant (either protective or causative) are highlighted in red. CI, Confidence Interval; N/A, not applicable. Klimisch Score adapted from *Regulatory Toxicology and Pharmacology* (1997) **25**, 1-5 [118].

**Additional file 3 Table S3.** Epidemiological studies investigating the association between snus use and stroke. Those epidemiological findings which are statistically significant (either protective or causative) are highlighted in red. N/A; not applicable. Klimisch Score adapted from *Regulatory Toxicology and Pharmacology* (1997) **25**, 1-5 [118].

**Additional file 4: Table S4.** Epidemiological studies investigating the association between snus use and pancreatic cancer. Those epidemiological findings which are statistically significant (either protective or causative) are highlighted in red. N/A; not applicable. Klimisch Score adapted from *Regulatory Toxicology and Pharmacology* (1997) **25**, 1-5 [118].

**Additional file 5: Table S5.** Epidemiological/clinical studies investigating the association between snus use and diabetes or metabolic syndrome. Those epidemiological findings which are statistically significant (either protective or causative) are highlighted in red. N/A; not applicable. Klimisch Score adapted from *Regulatory Toxicology and Pharmacology* (1997) **25**, 1-5 [118].

**Additional file 6: Table S6.** Epidemiological/clinical studies investigating the association between snus use and oral cancer. Those epidemiological findings which are statistically significant (either protective or causative) are highlighted in red. CI, Confidence Interval; N/A, not applicable. Klimisch Score adapted from *Regulatory Toxicology and Pharmacology* (1997) **25**, 1-5 [118].

**Additional file 7: Table S7.** Epidemiological/clinical studies investigating the association between snus use and periodontal disease and/or gingival disease. Those epidemiological findings which are statistically significant (either protective or causative) are highlighted in red. N/A; not applicable. Klimisch Score adapted from *Regulatory Toxicology and Pharmacology* (1997) **25**, 1-5 [118].

**Additional file 8: Table S8.** Epidemiological/clinical studies investigating the association between snus use and tooth loss and dental caries. Those epidemiological findings which are statistically significant (either protective or causative) are highlighted in red. N/A; not applicable. Klimisch Score adapted from *Regulatory Toxicology and Pharmacology* (1997) **25**, 1-5 [118].

## Data Availability

Not applicable.

## Abbreviations

BMI: Body mass index
CI: Confidence interval
EU: European Union
FDA: Food and Drug Administration
HR: Hazard ratio
MRTP: Modified risk tobacco product
OR: Odds ratio
RR: Relative risk
SCHENIR: Scientific Committee on Emerging and Newly Identified Health Risks
WHO: World Health Organisation
