# Supplementary material for: Snus: a compelling harm reduction alternative to cigarettes
Source: Harm Reduct J. 2019 Nov 27;16:62. doi: 10.1186/s12954-019-0335-1 (PMC6882181; doi:10.1186/s12954-019-0335-1)
Supplement: Supplementary file 1 — Additional file 1: Table S1. Epidemiological studies investigating the association between snus use and lung cancer. Those epidemiological findings which are statistically significant (either protective or causative) are highlighted in red. N/A; not applicable. Klimisch Score adapted from Regulatory Toxicology and Pharmacology (1997) 25, 1-5 [118]. [file 12954_2019_335_MOESM1_ESM.docx]

| Study | Epidemiological Findings | | | | |
| --- | --- | --- | --- | --- | --- |
| Boffetta et al., 2005 [ref. 34] |  | **Number of cases** | **Relative Risk (adjusted for age and smoking of cigarettes, cigars and pipe for stratification by snus use status; adjusted for age, and among current smokers, for amount of tobacco smoking for smoking status)** | **95% Confidence Interval** | **Scoring assessment of quality of the study**  **(based on assessment using the Klimisch Score)** |
|  | **Lung cancer (all histological types)**  Never Users [of snus]  Ever Users  Former Users  Current Users  **Lung cancer (adenocarcinoma)**  Never Users [of snus]  Ever Users  Former Users  Current Users  Never Smokers [of cigarettes]  Former Smokers  Current Smokers | 271  72  28  44  39  11  4  7  3  7  62 | REFERENCE  0.80  0.80  0.80  REFERENCE  0.83  0.86  0.81  0.96  0.64  0.68 | N/A  0.61-1.05  0.54-1.19  0.58-1.11  N/A  0.42-1.65  0.30-2.43  0.36-1.85  0.26-3.56  0.24-1.68  0.51-0.90 | 1 |
| Luo et al., 2007 [ref. 33]^1^ |  | **Number of cases** | **Relative Risk** | **95% Confidence Interval** | **Scoring assessment of quality of the study**  **(based on assessment using the Klimisch Score)** |
|  | Never Users [of any tobacco products]  Ever Users [of snus]  Former Users  Current Users  Amount Consumed (g/day)  1-9  ≥10 | 136  18  3  15  7  10 | REFERENCE  0.8  0.9  0.8  1.0  0.7 | N/A  0.5-1.3  0.3-5.0  0.4-1.3  0.5-2.1  0.4-1.3 | 1 |

**Supplementary Table 1**: Epidemiological studies investigating the association between snus use and lung cancer. Those epidemiological findings which are statistically significant (either protective or causative) are highlighted in red. N/A; not applicable. Klimisch Score adapted from *Regulatory Toxicology and Pharmacology* (1997) **25**, 1-5 [118].

^1^When the data for this study is analysed to include all cohort members, irrespective of smoking and snus use, the adjusted relative risk for lung cancer in ever users of snus compared with never users is 0.7 (0.6-0.7).
