# Supplementary material for: Snus: a compelling harm reduction alternative to cigarettes
Source: Harm Reduct J. 2019 Nov 27;16:62. doi: 10.1186/s12954-019-0335-1 (PMC6882181; doi:10.1186/s12954-019-0335-1)
Supplement: Supplementary file 2 — Additional file 2: Table S2. Epidemiological studies investigating the association between snus use and myocardial infarction. Those epidemiological findings which are statistically significant (either protective or causative) are highlighted in red. CI, Confidence Interval; N/A, not applicable. Klimisch Score adapted from Regulatory Toxicology and Pharmacology (1997) 25, 1-5 [118]. [file 12954_2019_335_MOESM2_ESM.docx]

| Study | Epidemiological Findings | | | | | | | | |
| --- | --- | --- | --- | --- | --- | --- | --- | --- | --- |
| Bolinder et al., 1994 [ref. 36] |  | **Number of cases** | **Relative Risk (adjusted for age (5-intervals) and for region of origin)** | | | **95% Confidence Interval** | | | **Scoring assessment of quality of the study**  **(based on assessment using the Klimisch Score)** |
|  | Non-Users  Smokeless Tobacco Users | 641  220 | REFERENCE  1.4 | | | N/A  1.2-1.6 | | | 2 [small number of cases of myocardial infarction in those individuals who use smokeless tobacco] |
| Hergens et al., 2007 [ref. 37]^1^ |  | **Number of cases** | **Relative Risk (adjusted for age, body mass index and region of residence)** | | | **95% Confidence Interval** | | | **Scoring assessment of quality of the study**  **(based on assessment using the Klimisch Score)** |
|  | **Age 35-54**  Never User  Ever User  Former User  Current User  **Age 55-65**  Never User  Ever User  Former User  Current User | 1,463  169  18  151  1,423  184  12  172 | **ALL CASES** | **NON-FATAL CASES** | **FATAL CASES** | **ALL CASES** | **NON-FATAL CASES** | **FATAL CASES** | 1 |
|  |  |  | REFERENCE  0.97  0.76  1.00  REFERENCE  1.04  0.69  1.08 | REFERENCE  0.90  0.63  0.94  REFERENCE  0.96  0.62  1.00 | REFERENCE  1.26  1.44  1.25  REFERENCE  1.21  0.87  1.26 | N/A  0.86-1.09  0.53-1.10  0.88-1.30  N/A  0.90-1.20  0.40-1.19  0.93-1.26 | N/A  0.79-1.04  0.41-0.98  0.82-1.09  N/A  0.80-1.15  0.31-1.23  0.83-1.21 | N/A  0.98-1.63  0.74-2.95  0.95-1.63  N/A  0.95-1.55  0.36-2.09  0.98-1.62 |  |
| Arefalk et al., 2012 [ref. 38] |  | **Number of cases** | **Hazard Ratio (^a^adjusted for age, current smoking dose, pack-years, diabetes, body mass index, occupation, alcohol use and myocardial infarction before baseline; ^b^adjusted for age, body mass index, region of residence, myocardial infarction before baseline)** | | | **95% Confidence Interval** | | | **Scoring assessment of quality of the study**  **(based on assessment using the Klimisch Score)** |
|  | Uppsala Longitudinal Study of Adult Men (n=1,076)^a^  Non-Users  Snus Users  Male Construction Workers (n=118, 425)^b^  Never tobacco use  Current Use  <12.5g/day  12.5-24.9g/day  25-49.9g/day  ≥50g/day  Former Snus Use | 81  14  464  75  28  35  8  4  6 | REFERENCE  **2.08**  REFERENCE  1.28  1.18  **1.46**  1.03  1.25  1.00 | | | N/A  **1.03-4.22**  REFERENCE  1.00-1.64  0.80-1.73  **1.03-2.06**  0.51-2.08  0.47-3.84  0.45-2.23 | | | 1 |
| Huhtasaari et al., 1992 [ref. 39] |  | **Number of cases** | **Relative Risk (adjusted for age with never users of any form of tobacco as the reference)** | | | **95% Confidence Interval** | | | **Scoring assessment of quality of the study**  **(based on assessment using the Klimisch Score)** |
|  | All Subjects  Age 35-54  Age 55-64 | 585 (cases); 589 (controls)  218 (cases); 396 (controls)  367 (cases); 193 (controls) | 0.89  0.96  1.24 | | | 0.62-1.29  0.56-1.67  0.67-1.29 | | | 2 [classification of never smokers included those individuals who reported occasional smoking] |
| Huhtasaari et al., 1999 [ref. 40]^2^ |  | **Number of cases** | **Odds Ratio** | | | **95% Confidence Interval** | | | **Scoring assessment of quality of the study**  **(based on assessment using the Klimisch Score)** |
|  | Never Users [of any tobacco]  Current Snus User with no smoking  Current Snus User with current smoking  Former Snus User with no smoking | 149 (cases); 217 (controls)  59 (cases); 90 (controls)  20 (cases); 11 (controls)  11 (cases); 13 (controls) | REFERENCE  0.96  **2.66**  1.23 | | | N/A  0.65-1.41  **1.24-5.71**  0.54-2.82 | | | 1 |

**Supplementary Table 2**: Epidemiological studies investigating the association between snus use and myocardial infarction. Those epidemiological findings which are statistically significant (either protective or causative) are highlighted in red. CI, Confidence Interval; N/A, not applicable. Klimisch Score adapted from *Regulatory Toxicology and Pharmacology* (1997) **25**, 1-5 [118].

^1^When results were further stratified by snus consumption (12.5, 12.5-24.9, 25-49.9 and ≥50g/day respectively), three statistically significant findings were observed: 12.5g/day consumption in those aged 35-54 for fatal cases (relative risk of 1.53, 95% CI 1.03-2.27), 12.5g/day consumption in those aged 55-65 for all cases (relative risk of 1.27, 95% CI 1.03-1.55) and ≥50g/day consumption in those aged 55-655 for fatal cases (relative risk of 2.46, 95% CI 1.09-5.55).

^2^When regular snus use was analysed in the absence of any other form of tobacco product use with respect to fatal and non-fatal cases (indicated as all cases and as fatal cases only), regular snus use was not shown to increase risk for either outcome [all cases combined, odds ratio of 0.58, 95% CI 0.35-0.94; fatal cases only, odds ratio 1.50, 95% CI 0.45-5.03].

| Study | Epidemiological Findings | | | | | | | | |
| --- | --- | --- | --- | --- | --- | --- | --- | --- | --- |
| Hergens et al., 2005 [ref. 41] |  | **Number of cases** | **Odds Ratio (adjusted for age, hospital catchment area and smoking)** | | | **95% Confidence Interval** | | | **Scoring assessment of quality of the study**  **(based on assessment using the Klimisch Score)** |
|  | Never Users  Former Users  Current Users | 1,432 (all cases)  1,173 (non-fatal cases)  259 (fatal cases) | **ALL CASES** | **NON-FATAL CASES** | **FATAL CASES** | **ALL CASES** | **NON-FATAL CASES** | **FATAL CASES** | 1 |
|  |  |  | REFERENCE  1.1  0.98 | REFERENCE  1.1  0.98 | REFERENCE  1.1  1.0 | N/A  0.78-1.5  0.77-1.3 | N/A  0.79-1.6  0.76-1.3 | N/A  0.54-2.1  0.65-1.6 |  |
| Haglund et al., 2007 [ref. 42] |  | **Number of cases** | **Incidence Rate Ratio (adjusted for age at event, socioeconomic status, residential area, self-reported health, number of longstanding illnesses and physical activity)** | | | **95% Confidence Interval** | | | **Scoring assessment of quality of the study**  **(based on assessment using the Klimisch Score)** |
|  | Never users [of tobacco]  Snus Users  Snus Users with current smoking | 227  28  15 | REFERENCE  0.77  1.64 | | | N/A  0.51-1.15  0.96-2.79 | | | 1 |
| Wennberg et al., 2007 [ref. 43]^3^ |  | **Number of cases** | **Odds Ratio (adjusted for body mass index, leisure time, physical activity, educational level and cholesterol level)** | | | **95% Confidence Interval** | | | **Scoring assessment of quality of the study**  **(based on assessment using the Klimisch Score)** |
|  | Never Users [of any tobacco]  Current Snus User with no smoking  Current Snus User with former smoking  Current smoker with no current snus use  Current Snus User with current smoking  Former Snus User with no smoking  Former smoker with no snus use  Former Snus User with former smoking | 130 (cases); 654 (controls)  21 (cases); 117 (controls)  37 (cases); 138 (controls)  136 (cases); 260 (controls)  30 (cases); 69 (controls)  11 (cases); 72 (controls)  58 (cases); 240 (controls)  33 (cases); 118 (controls) | REFERENCE  0.82  1.25  **2.60**  **2.14**  0.66  1.18  1.34 | | | N/A  0.46-1.43  0.80-1.96  **1.91-3.54**  **1.28-3.60**  0.32-1.34  0.82-1.70  0.84-2.12 | | | 1 |
| Janzon and Hedblad, 2009 [ref. 44] |  | **Number of cases** | **Relative Risk (adjusted for age, body mass index, smoking, diabetes, hypertension, physical activity, marital status and occupation)** | | | **95% Confidence Interval** | | | **Scoring assessment of quality of the study**  **(based on assessment using the Klimisch Score)** |
|  | Never Smokers  Former Smokers  Current Smokers | 118 (4 in current snus users)  195 (15 in current snus users)  231 (14 in current snus users) | 0.75  0.81  1.31 | | | 0.3-1.8  0.5-1.2  0.8-2.0 | | | 1 |
| Hansson et al., 2009 [ref. 45] |  | **Number of cases** | **Relative Risk (adjusted for age, smoking status (current or former), diabetes, high blood pressure and high cholesterol)** | | | **95% Confidence Interval** | | | **Scoring assessment of quality of the study**  **(based on assessment using the Klimisch Score)** |
|  | Never Users [of snus]  Ever Users  Former Users  Current Users  Amount Consumed (cans/week)  ≤4  >4  Duration (years)  <20  ≥20 | 630  130  60  70  55  14  22  47 | REFERENCE  0.98  1.17  0.86  0.84  0.92  0.87  0.85 | | | N/A  0.79-1.22  0.87-1.58  0.66-1.14  0.62-1.13  0.52-1.63  0.55-1.38  0.62-1.18 | | | 1 |
| Hansson et al., 2012 [ref. 46] |  | **Number of cases** | **Hazard Ratio (adjusted for age and body mass index)** | | | **95% Confidence Interval** | | | **Scoring assessment of quality of the study**  **(based on assessment using the Klimisch Score)** |
|  | Non-current Use^4^  Current use (cans/week)  <4  4-6  ≥7  Non-current Use^4^  Duration (years)  <20  ≥20 | 2,873  270  27  26  2,580  136  200 | REFERENCE  1.02  0.94  1.17  REFERENCE  0.96  1.10 | | | N/A  0.90-1.16  0.64-1.38  0.79-1.72  N/A  0.80-1.14  0.95-1.27 | | | 1 |

**Supplementary Table 2 (continued)**: Epidemiological studies investigating the association between snus use and myocardial infarction. Those epidemiological findings which are statistically significant (either protective or causative) are highlighted in red. N/A; not applicable. Klimisch Score adapted from *Regulatory Toxicology and Pharmacology* (1997) **25**, 1-5 [118].

^3^Further analyses regarding the risk of fatal myocardial infarction within 28 days and sudden cardiac death with a survival time of either less than one or twenty-four hours showed similar findings with the only statistically significant increase in risk observed for current cigarette smokers (with no current snus use).

^4^Non-current use constitutes the reference group, rather than never-users. Analysis restricted to never smokers of cigarettes to remove smoking as a confounding factor.
