# Supplementary material for: Snus: a compelling harm reduction alternative to cigarettes
Source: Harm Reduct J. 2019 Nov 27;16:62. doi: 10.1186/s12954-019-0335-1 (PMC6882181; doi:10.1186/s12954-019-0335-1)
Supplement: Supplementary file 3 — Additional file 3 Table S3. Epidemiological studies investigating the association between snus use and stroke. Those epidemiological findings which are statistically significant (either protective or causative) are highlighted in red. N/A; not applicable. Klimisch Score adapted from Regulatory Toxicology and Pharmacology (1997) 25, 1-5 [118]. [file 12954_2019_335_MOESM3_ESM.docx]

| Study | Epidemiological Findings | | | | | | | | | | | | | |
| --- | --- | --- | --- | --- | --- | --- | --- | --- | --- | --- | --- | --- | --- | --- |
| Bolinder et al., 1994 [ref. 36] |  | **Number of cases** | | | | **Relative Risk (adjusted for age (5-intervals), and for region of origin, blood pressure, blood pressure medication, previous cardiac symptoms [unspecified], diabetes and body mass index)** | | | | **95% Confidence Interval** | | | | **Scoring assessment of quality of the study**  **(based on assessment using the Klimisch Score)** |
|  | **Age at entry into study**  **35-54**  Non-Users  Smokeless Tobacco Users  **55-65**  Non-Users  Smokeless Tobacco Users | 16  4  70  26 | | | | REFERENCE  1.9  REFERENCE  1.2 | | | | N/A  0.6-5.7  N/A  0.7-1.8 | | | | 2 [extremely small number of cases of strokes in those individuals who use smokeless tobacco] |
| Asplund et al., 2003 [ref. 31] |  | **Number of cases** | | | | **Relative Risk** | | | | **95% Confidence Interval** | | | | **Scoring assessment of quality of the study**  **(based on assessment using the Klimisch Score)** |
|  | Never Users [of tobacco]  Snuff Users | 85 [of 276 cases]; 210 [of 551 controls]  30 [of 276 cases]; 65 [of 551 controls] | | | | REFERENCE  1.16 | | | | N/A  0.60-2.22 | | | | 2 [those categorised as being snuff users includes those who also reported as being former conventional cigarette smokers] |
| Haglund et al., 2007 [ref. 42] |  | **Number of cases** | | | | **Incidence Rate Ratio (adjusted for age at event, socioeconomic status, residential area, self-reported health, number of longstanding illnesses and physical activity)** | | | | **95% Confidence Interval** | | | | **Scoring assessment of quality of the study**  **(based on assessment using the Klimisch Score)** |
|  | Never users [of tobacco]  Snus Users  Snus Users with current smoking | 126  19  9 | | | | REFERENCE  1.07  1.98 | | | | N/A  0.65-1.77  1.00-3.95 | | | | 1 |
| Hergens et al., 2008 [ref. 48] |  | **Number of cases** | | | | **Relative Risk (adjusted for age, body mass index and region of residence)** | | | | **95% Confidence Interval** | | | | **Scoring assessment of quality of the study**  **(based on assessment using the Klimisch Score)** |
|  | **ALL STROKE TYPES**  All  Nonfatal  Fatal  **ISCHAEMIC STROKE**  All  Nonfatal  Fatal  **HAEMORRHAGIC STROKE**  All  Nonfatal  Fatal  **UNSPECIFIED STROKE**  All  Nonfatal  Fatal | Never-Users | Ever Users | Former Users | Current Users | Never-Users | Ever Users | Former Users | Current Users | Never-Users | Ever Users | Former Users | Current Users | 1 |
|  |  | 2,805  2,569  236  1,979  1,887  92  474  378  96  352  304  48 | 443  398  45  304  282  22  76  60  16  63  56  7 | 31  30  1  20  19  1  8  8  0  3  3  0 | 412  161  21  129  118  11  26  19  7  28  24  4 | REFERENCE REFERENCE  REFERENCE  REFERENCE  REFERENCE  REFERENCE  REFERENCE  REFERENCE  REFERENCE  REFERENCE  REFERENCE  REFERENCE | 1.02  1.00  1.27  1.03  1.00  **1.63**  0.86  0.82  1.05  1.22  1.25  1.03 | 0.72  0.75  0.30  0.68  0.67  0.82  0.90  1.10  N/A  0.66  0.69  N/A | 1.05  1.02  1.38  1.07  1.04  **1.72**  0.85  0.77  1.17  **1.35**  1.31  1.14 | N/A  N/A  N/A  N/A  N/A  N/A  N/A  N/A  N/A  N/A  N/A  N/A | 0.92-1.13  0.89-1.11  0.92-1.76  0.91-1.16  0.88-1.13  **1.02-1.62**  0.67-1.10  0.62-1.08  0.61-1.80  0.93-1.61  0.93-1.67  0.47-2.31 | 0.50-1.02  0.53-1.08  0.04-2.11  0.44-1.06  0.43-1.06  0.12-5.93  0.45-1.82  0.54-2.21  N/A  0.21-2.06  0.22-2.14  N/A | 0.95-1.17  0.91-1.14  0.99-1.91  0.94-1.22  0.91-1.18  **1.06-2.78**  0.65-1.10  0.57-1.04  0.68-2.01  **1.02-1.80**  0.98-1.77  0.51-2.54 |  |
| Janzon and Hedblad, 2009 [ref. 44] |  | **Number of cases** | | | | **Relative Risk (adjusted for age, body mass index, smoking, diabetes, hypertension, physical activity, marital status and occupation)** | | | | **95% Confidence Interval** | | | | **Scoring assessment of quality of the study**  **(based on assessment using the Klimisch Score)** |
|  | Never Smokers  Former Smokers  Current Smokers | 140 (4 in current snus users)  229 (18 in current snus users)  184 (13 in current snus users) | | | | 0.59  0.88  1.13 | | | | 0.2-1.5  0.5-1.4  0.6-2.0 | | | | **1** |

**Supplementary Table 3**: Epidemiological studies investigating the association between snus use and stroke. Those epidemiological findings which are statistically significant (either protective or causative) are highlighted in red. N/A; not applicable. Klimisch Score adapted from *Regulatory Toxicology and Pharmacology* (1997) **25**, 1-5 [118].

| Study | Epidemiological Findings | | | | |
| --- | --- | --- | --- | --- | --- |
| Hansson et al., 2009 [ref. 45] |  | **Number of cases** | **Relative Risk (adjusted for age, smoking status (current or former), diabetes, high blood pressure and high cholesterol)** | **95% Confidence Interval** | **Scoring assessment of quality of the study**  **(based on assessment using the Klimisch Score)** |
|  | Never Users [of snus]  Ever Users  Former Users  Current Users  Amount Consumed (cans/week)  ≤4  >4  Duration (years)  <20  ≥20 | 351  65  29  36  24  12  13  22 | REFERENCE  1.01  1.17  0.91  0.75  1.75  1.13  0.80 | N/A  0.76-1.34  0.78-1.74  0.64-1.31  0.49-1.15  0.95-3.21  0.63-2.01  0.51-1.25 | 1 |
| Hansson et al., 2014 [ref. 49] |  | **Number of cases** | **Hazard Ratio (adjusted for age and body mass index)** | **95% Confidence Interval** | **Scoring assessment of quality of the study**  **(based on assessment using the Klimisch Score)** |
|  | **ALL STROKE TYPES**  Amount Consumed (cans/week)  <4  4-6  ≥7  Duration (years)  <20  ≥20  **ISCHAEMIC STROKE**  Amount Consumed (cans/week)  <4  4-6  ≥7  Duration (years)  <20  ≥20  **HAEMORRHAGIC STROKE**  Amount Consumed (cans/week)  <4  4-6  ≥7  Duration (years)  <20  ≥20  **UNSPECIFIED STROKE**  Amount Consumed (cans/week)  <4  4-6  ≥7  Duration (years)  <20  ≥20 | 235  26  14  130  152  151  16  6  76  103  52  8  5  44  24  32  2  3  10  25 | 1.05  1.00  0.72  0.98  1.05  1.06  1.02  0.54  1.01  1.05  0.95  1.02  0.78  0.99  0.89  1.16  0.75  1.52  0.79  1.26 | 0.92-1.20  0.67-1.47  0.42-1.22  0.81-1.18  0.89-1.23  0.89-1.26  0.62-1.68  0.24-1.26  0.79-1.29  0.85-1.28  0.71-1.27  0.51-2.07  0.32-1.90  0.71-1.38  0.59-1.35  0.81-1.68  0.19-3.01  0.49-4.79  0.41-1.51  0.83-1.89 | 1 |

**Supplementary Table 3 (continued)**: Epidemiological studies investigating the association between snus use and stroke. Those epidemiological findings which are statistically significant (either protective or causative) are highlighted in red. N/A; not applicable. Klimisch Score adapted from *Regulatory Toxicology and Pharmacology* (1997) **25**, 1-5 [118].
