# Supplementary material for: Snus: a compelling harm reduction alternative to cigarettes
Source: Harm Reduct J. 2019 Nov 27;16:62. doi: 10.1186/s12954-019-0335-1 (PMC6882181; doi:10.1186/s12954-019-0335-1)
Supplement: Supplementary file 4 — Additional file 4: Table S4. Epidemiological studies investigating the association between snus use and pancreatic cancer. Those epidemiological findings which are statistically significant (either protective or causative) are highlighted in red. N/A; not applicable. Klimisch Score adapted from Regulatory Toxicology and Pharmacology (1997) 25, 1-5 [118]. [file 12954_2019_335_MOESM4_ESM.docx]

| Study | Epidemiological Findings | | | | |
| --- | --- | --- | --- | --- | --- |
| Boffetta et al., 2005 [ref. 32] |  | **Number of cases** | **Relative Risk (adjusted for age and smoking of cigarettes, cigars and pipe for stratification by snus use status; adjusted for age, and among current smokers, for amount of tobacco smoking for smoking status)** | **95% Confidence Interval** | **Scoring assessment of quality of the study**  **(based on assessment using the Klimisch Score)** |
|  | Never Users [of snus]  Ever Users  Former Users  Current Users  Never Smokers [of cigarettes]  Former Smokers  Current Smokers | 60  45  18  27  3  14  28 | REFERENCE  **1.67**  **1.80**  1.60  0.85  1.37  **1.86** | N/A  **1.12-2.50**  **1.04-3.09**  1.00-2.55  0.24-3.07  0.59-3.17  **1.13-3.05** | 2 [no adjustment for alcohol consumption or diabetes] |
| Luo et al., 2007 [ref. 31]^1^ |  | **Number of cases** | **Relative Risk (adjusted for attained age and body mass index)** | **95% Confidence Interval** | **Scoring assessment of quality of the study**  **(based on assessment using the Klimisch Score)** |
|  | Never Users [of any tobacco products]  Ever Users [of snus]  Former Users  Current Users  Amount Consumed (g/day)  1-9  ≥10 | 63  20  2  18  6  13 | REFERENCE  **2.0**  1.4  **2.1**  1.9  **2.1** | N/A  **1.2-3.3**  0.4-5.9  **1.2-3.6**  0.8-4.3  **1.1-3.8** | 2 [no adjustment for alcohol consumption or diabetes] |
| Araghi et al., 2017 [ref. 64] |  | **Number of cases** | **Hazard ratio (adjusted for attained age, smoking status and body mass index)** | **95% Confidence Interval** | **Scoring assessment of quality of the study**  **(based on assessment using the Klimisch Score)** |
|  | Never Users [of snus]  Ever Users  Former Users  Current Users  Amount Consumed (cans/week) [current users only]  <4  4-6  ≥7  Duration (years)  [current users only]  ≤5  5-<10  10-<15  15-<20  ≥20 | 1,103  321  93  227  91  83  48  27  38  41  27  78 | REFERENCE  0.93  0.88  0.96  0.87  1.16  0.87  0.82  1.00  0.99  0.98  0.95 | N/A  0.82-1.06  0.71-1.10  0.83-1.11  0.70-1.08  0.93-1.46  0.65-1.17  0.56-1.21  0.72-1.39  0.72-1.36  0.67-1.44  0.75-1.19 | 1 |

**Supplementary Table 4**: Epidemiological studies investigating the association between snus use and pancreatic cancer. Those epidemiological findings which are statistically significant (either protective or causative) are highlighted in red. N/A; not applicable. Klimisch Score adapted from *Regulatory Toxicology and Pharmacology* (1997) **25**, 1-5 [118].

^1^When the data for this study is analysed to include all cohort members, irrespective of smoking and snus use, the adjusted relative risk for pancreatic cancer in ever users of snus compared with never users is 0.9 (0.7-1.2).
