# Supplementary material for: Snus: a compelling harm reduction alternative to cigarettes
Source: Harm Reduct J. 2019 Nov 27;16:62. doi: 10.1186/s12954-019-0335-1 (PMC6882181; doi:10.1186/s12954-019-0335-1)
Supplement: Supplementary file 5 — Additional file 5: Table S5. Epidemiological/clinical studies investigating the association between snus use and diabetes or metabolic syndrome. Those epidemiological findings which are statistically significant (either protective or causative) are highlighted in red. N/A; not applicable. Klimisch Score adapted from Regulatory Toxicology and Pharmacology (1997) 25, 1-5 [118]. [file 12954_2019_335_MOESM5_ESM.docx]

| Study | Epidemiological/Clinical Findings | | | | |
| --- | --- | --- | --- | --- | --- |
| Eliasson et al., 1991 [ref. 58] |  | **Number of participants** | **Clinical Findings** | **95% Confidence Interval** | **Scoring assessment of quality of the study**  **(based on assessment using the Klimisch Score)** |
|  |  | Current snus users, n=21; non-tobacco users, n=18; conventional cigarette smokers, n=19. All participants were young men of comparable age and body mass index. | Both groups of tobacco-users showed increased serum insulin levels (5.5 and 8.6mU/L for snus and conventional cigarette smokers respectively) compared to the control group (3.6mU/L) at similar blood glucose concentrations (4.3-4.4mmol/L for all groups). In contrast to the conventional cigarette smokers, snus users showed no significant elevation of diastolic blood pressure, haemoglobin concentrations, white cell count, serum cholesterol or triglyceride levels. | N/A | 2 [a small number of participants in study] |
| Eliasson et al., 1995 [ref. 60] |  | **Number of participants** | **Clinical Findings** | **95% Confidence Interval** | **Scoring assessment of quality of the study**  **(based on assessment using the Klimisch Score)** |
|  |  | Total cohort, n=1,266 (men, n=604; women, n=662). Aged between 25 and 64 years. Non-tobacco users, n=581; former smokers, n=238; current smokers, n=317; snus users, n=104; dual users, n=42. Snus use only present and analysed in men. | Fasting and post-load insulin, fibrinogen, tissue plasminogen and plasminogen activator inhibitor type (PAI-1) levels were not significantly affected by snus use. Levels were significantly perturbed by conventional cigarette use. | N/A | 1 |
| Norberg et al., 2006 [ref.63] |  | **Number of cases** | **Odds Ratio (adjusted for age, gender, family of history or cardiovascular disease and/or diabetes in first-degree relatives at follow-up)** | **95% Confidence Interval** | **Scoring assessment of quality of the study**  **(based on assessment using the Klimisch Score)** |
|  | **METABOLIC SYNDROME**  No snus Use  Snus Use, ≤4 cans/week  Snus Use, >4 cans/week  **DIABETES**  No snus Use  Snus Use, ≤4 cans/week  Snus Use, >4 cans/week  **ELEVATED TRIGLYCERIDES**  No snus Use  Snus Use, ≤4 cans/week  Snus Use, >4 cans/week  **LOW HDL CHOLESTEROL**  No snus Use  Snus Use, ≤4 cans/week  Snus Use, >4 cans/week  **HYPERTENSION**  No snus Use  Snus Use, ≤4 cans/week  Snus Use, >4 cans/week  **ELEVATED BODY MASS INDEX (≥30kg/m^2^)**  No snus Use  Snus Use, ≤4 cans/week  Snus Use, >4 cans/week | 1,498 (cases); 12,344 (controls)  174 (cases); 1,516 (controls)  74 (cases); 396 (controls) | REFERENCE  1.0  **1.6**  REFERENCE  1.0  0.8  REFERENCE  **1.2**  **1.6**  REFERENCE  1.0  1.1  REFERENCE  0.9  1.2  REFERENCE  1.0  **1.7** | N/A  0.85-1.22  **1.26-2.15**  N/A  0.86-1.08  0.69-1.02  N/A  **1.05-1.35**  **1.30-1.95**  N/A  0.86-1.18  0.82-1.42  N/A  0.84-1.05  0.99-1.46  N/A  0.88-1.20  **1.36-2.18** | 1 |

**Supplementary Table 5**: Epidemiological/clinical studies investigating the association between snus use and diabetes or metabolic syndrome. Those epidemiological findings which are statistically significant (either protective or causative) are highlighted in red. N/A; not applicable. Klimisch Score adapted from *Regulatory Toxicology and Pharmacology* (1997) **25**, 1-5 [118].

| Study | Epidemiological/Clinical Findings | | | | |
| --- | --- | --- | --- | --- | --- |
| Byhamre et al., 2017 [ref. 64] |  | **Number of cases** | **Odds Ratio (adjusted for gender, cumulative smoking, body mass index at 16, socioeconomic status at 16, family history of diabetes, alcohol consumption at 43 and physical activity at 43**) | **95% Confidence Interval** | **Scoring assessment of quality of the study**  **(based on assessment using the Klimisch Score)** |
|  | **METABOLIC SYNDROME**  Age 16 (1981)  Age 21 (1986)  Age 30 (1995)  Age 43 (2008)  **CENTRAL OBESITY**  Age 16 (1981)  Age 21 (1986)  Age 30 (1995)  Age 43 (2008)  **RAISED TRIGLYCERIDES**  Age 16 (1981)  Age 21 (1986)  Age 30 (1995)  Age 43 (2008)  **LOW HDL-C**  Age 16 (1981)  Age 21 (1986)  Age 30 (1995)  Age 43 (2008)  **HIGH BLOOD PRESSURE**  Age 16 (1981)  Age 21 (1986)  Age 30 (1995)  Age 43 (2008)  **IMPAIRED FASTING GLUCOSE OR TYPE II DIABETES**  Age 16 (1981)  Age 21 (1986)  Age 30 (1995)  Age 43 (2008) | 81  53  57  37  81  53  57  37  81  53  57  37  81  53  57  37  81  53  57  37  81  53  57  37 | 0.95  1.15  1.01  1.15  1.40  1.24  1.15  1.65  1.38  1.27  1.37  1.10  1.23  0.84  0.53  0.69  1.08  1.31  1.61  1.41  1.08  1.28  1.01  0.38 | 0.54-1.65  0.60-2.21  0.52-1.99  0.52-2.51  0.83-2.35  0.65-2.34  0.61-2.15  0.76-3.58  0.81-2.37  0.66-2.45  0.71-2.63  0.49-2.45  0.72-2.12  0.41-1.70  0.25-1.12  0.29-1.66  0.66-1.77  0.71-2.42  0.88-2.96  0.69-2.89  0.59-1.97  0.63-2.62  0.48-2.11  0.12-1.16 | 1 |
| Persson et al., 2000 [ref.68] |  | **Number of cases/controls** | **Relative Risk (adjusted for age, body mass index, family history of diabetes, physical activity and alcohol consumption)** | **95% Confidence Interval** | **Scoring assessment of quality of the study**  **(based on assessment using the Klimisch Score)** |
|  | Never Users [of snus]  Former Users  Current Users  Consumption (boxes per week;  current users only)  Never  ≤2  3+  Current tobacco use  Never  Snus Only  Conventional cigarettes only | 1,1915 controls; 34 cases  376 controls; 5 cases  492 controls; 13 cases  1,915 controls; 34 cases  235 controls, 1 case  256 controls; 12 cases  895 controls; 9 cases  121 controls; 4 cases  517 controls; 14 cases | REFERENCE  0.8  1.5  REFERENCE  0.2  **2.7**  REFERENCE  **3.9**  1.8 | N/A  0.3-2.0  0.8-3.0  N/A  0.0-2.0  **1.3-5.5**  N/A  **1.1-14.3**  0.7-4.5 | 2 [statistically significant observations based on small number cases of Type II diabetes and with wide 95% confidence intervals] |
| Eliasson et al., 2004 [ref. 69] |  | **Number of subjects [at baseline]** | **Odds ratio (adjusted for age, follow-up, annual percentage weight gain between baseline and follow-up)** | **95% Confidence Interval** | **Scoring assessment of quality of the study**  **(based on assessment using the Klimisch Score)** |
|  | No Tobacco Use  Consistent exclusive snus users  Consistent exclusive smokers  Former smokers  Former snus users  Smokers who switched to snus | 1,203  475  889  475  161  **[number not reported]** | REFERENCE  N/A [no cases reported]  **4.61**  **3.13**  1.72  3.25 | N/A  N/A  **1.37-15.5**  **1.13-8.67**  0.20-14.8  0.78-13.6 | 2 [no data available for current snus users as no cases of Type II diabetes reported for these individuals; data only available on former snus use] |

**Supplementary Table 5**: Epidemiological/clinical studies investigating the association between snus use and diabetes or metabolic syndrome. Those epidemiological findings which are statistically significant (either protective or causative) are highlighted in red. N/A; not applicable. Klimisch Score adapted from *Regulatory Toxicology and Pharmacology* (1997) **25**, 1-5 [118].

| Study | Epidemiological/Clinical Findings | | | | |
| --- | --- | --- | --- | --- | --- |
| Wändell et al., 2008 [ref. 70]^1^ |  | **Number of participants in cohort (total cohort, n=1,859; men aged 60 years)** | **Odds Ratio** | **95% Confidence Interval** | **Scoring assessment of quality of the study**  **(based on assessment using the Klimisch Score)** |
|  | **METABOLIC SYNDROME**  Never users of tobacco  Current snus users  Former Snus users  Current snus users, former smokers  Dual Users  **NEWLY DIAGNOSED DIABETES**  Never users of tobacco  Current snus users  Former Snus users  Current snus users, former smokers  Dual Users  Snus, low consumers (<3 cans per week)  Snus, high consumers (≥3 cans per week) | 594  16  12  113  27  594  16  12  113  27  62%  38% | REFERENCE  1.55  0.69  1.14  1.46  REFERENCE  2.12  3.10  1.71  2.48  1.30  1.80 | N/A  0.52-4.62  0.14-3.28  0.71-1.82  0.63-3.41  N/A  0.25-17.71  0.36-26.84  0.67-4.35  0.52-11.82  0.49-3.40  0.67-4.85 | 1 |
| Neumann et al., 2013 [ref. 71] |  | **Number of participants in cohort** | **Odds Ratio** | **95% Confidence Interval** | **Scoring assessment of quality of the study**  **(based on assessment using the Klimisch Score)** |
|  | No Current Use  ≤4 cans per week  >4 cans per week  Data Missing | 24,927  3,293  973  744 | REFERENCE  Normal Glucose Tolerance to Impaired Fasting Glucose: 0.92  Normal Glucose Tolerance to Impaired Fasting Glucose and Impaired Glucose Tolerance: 0.79 | N/A  0.82-1.03  0.59-1.05 | 1 |
| Rasouli et al., 2017 [ref. 72] |  | **Number of cases/controls** | **Odds Ratio (adjusted for age, smoking, body mass index and family history of diabetes)** | **95% Confidence Interval** | **Scoring assessment of quality of the study**  **(based on assessment using the Klimisch Score)** |
|  | **ESTRID STUDY, 2010-2015**  Never snus use  Former snus use  Current snus use  Boxes per week (ever snus users only)  Never  <5 boxes per week  ≥5 boxes per week  Box-years (ever snus users only)  Never  <10  ≥10  **HUNT3 SURVEY, 2006-2008**  Never snus use  Ever snus use  Boxes per week (ever snus users only)  Never  35 boxes per week  ≥3 boxes per week | 515 (cases); 477 (controls)  80 (cases); 89 (controls)  129 (cases); 133 (controls)  515 (cases); 477 (controls)  143 (cases); 161 (controls)  62 (cases); 55 (controls)  390 (cases); 388 (controls)  92 (cases); 123 (controls)  68 (cases); 55 (controls)  672 (cases); 14,826 (controls)  157 (cases); 5,777 (controls)  669 (cases); 14,804 (controls)  130 (cases); 5,073 (controls)  9 (cases); 438 (controls) | REFERENCE  0.63  0.96  REFERENCE  0.78  0.95  REFERENCE  0.74  1.05  REFERENCE  0.91  REFERENCE  0.88  0.92 | N/A  0.41-0.95  0.67-1.37  N/A  0.56-1.09  0.57-1.58  N/A  0.52-1.06  0.67-1.63  N/A  0.75-1.10  N/A  0.72-1.08  0.46-1.83 | 1 |
| Östenson et al., 2012 [ref. 73] |  | **Number of cases/controls** | **Odds ratio (adjusted for age, body mass index, glucose tolerance, physical activity, alcohol consumption, socioeconomic position, family history of diabetes and smoking)** | **95% Confidence Interval** | **Scoring assessment of quality of the study**  **(based on assessment using the Klimisch Score)** |
|  | Consistent never snus use  Consistent snus use  Former snus use  Consistent never snus use  1-5 boxes per week (consistent users)  >5 boxes per week (consistent users) | 64 (cases); 1,367 (controls)  16 (cases); 285 (controls)  6 (cases); 207 (controls)  64 (cases); 1,367 (controls)  7 (cases); 226 (controls)  9 (cases); 59 (controls) | REFERENCE  1.1  0.5  REFERENCE  0.6  **3.3** | N/A  0.6-2.0  0.2-1.2  N/A  0.2-1.4  **1.4-8.1** | 1 |

**Supplementary Table 5**: Epidemiological/clinical studies investigating the association between snus use and diabetes or metabolic syndrome. Those epidemiological findings which are statistically significant (either protective or causative) are highlighted in red. N/A; not applicable. Klimisch Score adapted from *Regulatory Toxicology and Pharmacology* (1997) **25**, 1-5 [118].

^1^Data presented for metabolic syndrome based on diagnosis using ATP III criteria. Findings based on assessment using two other sets of criteria (EGIR and IDF) show comparable results. Data presented for newly diagnosed diabetes obtained using two different models.

| Study | Epidemiological/Clinical Findings | | | | |
| --- | --- | --- | --- | --- | --- |
| Carlsson et al., 2017 [ref. 74] |  | **Number of cases** | **Hazard Ratio (adjusted for age, calendar year, body mass index, physical activity, education and alcohol consumption)** | **95% Confidence Interval** | **Scoring assessment of quality of the study**  **(based on assessment using the Klimisch Score)** |
|  | Never Users  Current Users  Former Users  Number of boxes per week  (current users only)  1-2  3-4  5-6  ≥7  1-4  ≥4  Duration of Use (years)  (current users only)  <30  ≥30 | 2,075  248  118  54  83  54  31  137  85  66  152 | REFERENCE  1.15  0.86  1.14  1.03  **1.42**  **1.68**  1.08  **1.43**  **1.34**  1.17 | N/A  1.00-1.32  0.71-1.05  0.86-1.50  0.82-1.29  **1.07-1.87**  **1.17-2.41**  0.90-1.29  **1.15-1.79**  **1.03-1.73**  0.98-1.39 | 1 |

**Supplementary Table 5**: Epidemiological/clinical studies investigating the association between snus use and diabetes or metabolic syndrome. Those epidemiological findings which are statistically significant (either protective or causative) are highlighted in red. N/A; not applicable. Klimisch Score adapted from *Regulatory Toxicology and Pharmacology* (1997) **25**, 1-5 [118].
