# Supplementary material for: Snus: a compelling harm reduction alternative to cigarettes
Source: Harm Reduct J. 2019 Nov 27;16:62. doi: 10.1186/s12954-019-0335-1 (PMC6882181; doi:10.1186/s12954-019-0335-1)
Supplement: Supplementary file 6 — Additional file 6: Table S6. Epidemiological/clinical studies investigating the association between snus use and oral cancer. Those epidemiological findings which are statistically significant (either protective or causative) are highlighted in red. CI, Confidence Interval; N/A, not applicable. Klimisch Score adapted from Regulatory Toxicology and Pharmacology (1997) 25, 1-5 [118]. [file 12954_2019_335_MOESM6_ESM.docx]

| Study | Epidemiological/Clinical Findings | | | | |
| --- | --- | --- | --- | --- | --- |
| Bundgaard et al., 1995 [ref. 80] |  | **Number of cases** | **Odds ratio (adjusted for lifetime consumption of tobacco and alcohol)** | **95% Confidence Interval** | **Scoring assessment of quality of the study**  **(based on assessment using the Klimisch Score)** |
|  | **Lifetime tobacco consumption^1^**  0  1-135kg  136-235kg  >236kg  **Current tobacco consumption**  0  1-20g/day  >21g/day | 25 (cases); 136 (controls)  30 (cases); 116 (controls)  32 (cases); 76 (controls)  73 (cases); 68 (controls)  43 (cases); 228 (controls)  58 (cases); 128 (controls)  52 (cases); 41 (controls) | REFERENCE  1.7  **2.5**  **6.3**  REFERENCE  **2.1**  **5.8** | N/A  0.9-3.2  **1.3-5.0**  **3.1-12.9**  N/A  **1.3-3.5**  **3.1-10.9** | 3 [study did not stratify the results by the type of tobacco used and none of the participants were reported to be current users of snus**]^2^** |
| Lewin et al., 1998 [ref. 77]^3^ |  | **Number of cases** | **Relative Risk (adjusted for age, geographical location, alcohol consumption and conventional cigarette smoking)** | **95% Confidence Interval** | **Scoring assessment of quality of the study**  **(based on assessment using the Klimisch Score)** |
|  | Never Users [of Snus]  Ever Users  Former Users  Current Users | 462  83  40  43 | REFERENCE  1.1  1.0  1.2 | N/A  0.7-1.5  0.6-1.6  0.7-1.9 | 1 |
| Schildt et al., 1998 [ref. 78] |  | **Number of cases/controls** | **Relative Risk (adjusted for age, geographical location, alcohol consumption and conventional cigarette smoking)** | **95% Confidence Interval** | **Scoring assessment of quality of the study**  **(based on assessment using the Klimisch Score)** |
|  | Never Users [of Snus]  Ever Users  Former Users  Current Users | 287/282  67/72  28/18  39/54 | REFERENCE  0.9  1.5  0.7 | N/A  0.6-1.4  0.8-2.9  0.4-1.1 | 1 |
| Boffetta et al., 2005 [ref. 34] |  | **Number of cases** | **Relative Risk (adjusted for age and smoking of cigarettes, cigars and pipe for stratification by snus use status; adjusted for age, and among current smokers, for amount of tobacco smoking for smoking status)** | **95% Confidence Interval** | **Scoring assessment of quality of the study**  **(based on assessment using the Klimisch Score)** |
|  | **Oral/pharyngeal cancer**  Never Users [of snus]  Ever Users  Former Users  Current Users | 25  9  3  6 | REFERENCE  1.10  1.04  1.13 | N/A  0.50-2.41  0.31-3.50  0.45-2.83 | 1 |
| Rosenquist et al., 2005 [ref. 79]^4^ |  | **Number of cases** | **Odds Ratio (adjusted for total consumption of alcohol and smoking of tobacco)** | **95% Confidence Interval** | **Scoring assessment of quality of the study**  **(based on assessment using the Klimisch Score)** |
|  | Never Users  Former Users  Current Users | 112 (cases); 255 (controls)  7 (cases); 34 (controls)  13 (cases); 31 (controls) | REFERENCE  0.3  1.1 | N/A  0.1-0.9  0.5-2.5 | 1 |
| Luo et al., 2007 [ref. 33] |  | **Number of cases** | **Relative Risk** | **95% Confidence Interval** | **Scoring assessment of quality of the study**  **(based on assessment using the Klimisch Score)** |
|  | Never Users [of any tobacco products]  Ever Users [of snus]  Former Users  Current Users  Amount Consumed (g/day)  1-9  ≥10 | 50  10  1  9  2  8 | REFERENCE  0.8  0.7  0.9  0.7  0.9 | N/A  0.4-1.7  0.1-5.0  0.4-1.8  0.2-2.8  0.4-2.0 | 2 [extremely small number of cases of oral cancers] |
| Roosaar et al., 2008 [ref 81]^5^ |  | **Number of cases** | **Hazard Ratio** | **95% Confidence Interval** | **Scoring assessment of quality of the study**  **(based on assessment using the Klimisch Score)** |
|  | Never Users [of snus]  Ever Users | 6  5 | REFERENCE  2.3 | N/A  0.7-8.3 | 2 [extremely small number of cases of oral and pharyngeal cancer in never smokers] |
| Hirsch et al., 2012 [82] |  | **Number of cases** | **CLINICAL FINDINGS** | **95% Confidence Interval** | **Scoring assessment of quality of the study**  **(based on assessment using the Klimisch Score)** |
|  |  | 16 | A case report series of sixteen male patients diagnosed with oral squamous cell carcinoma. Mean age of approximately 73 years. Mean duration of snus use prior to cancer diagnosis was approximately 43 years. Ten patients were exclusive snus users, five were also former smokers and one patient was a dual user. | N/A | 2 [conventional cigarette use is a significant confounding factor in approximately one third of cases. No adjustment for alcohol use] |

**Supplementary Table 6**: Epidemiological/clinical studies investigating the association between snus use and oral cancer. Those epidemiological findings which are statistically significant (either protective or causative) are highlighted in red. CI, Confidence Interval; N/A, not applicable. Klimisch Score adapted from *Regulatory Toxicology and Pharmacology* (1997) **25**, 1-5 [118].

^1^One gram of tobacco assumed to equal one cigarette.

^2^None of the participants in either group were current users of snus and “*very few individuals*” had a previous history of snuff use. The authors of this study concluded that “*from the figures that chewing tobacco and snuff [snus] hardly can have played any quantitatively significant role in the development of intra-oral carcinoma in Denmark*” (p64).

^3^Further analyses indicated no statistically significant effect on risk of age at which snus use was initiated, duration of use, total consumption, intensity of use or histological site of cancer (oral cavity, larynx, oesophagus and pharynx).

^4^Further analyses indicated no statistically significant effect on risk when results were stratified based on whether or not the snus was fermented, by duration of use (years), exposure time per day (h/day) or by consumption per day (g/day).

^5^When analyses included all participants in the cohort [and was restricted to never smokers only], a statistically significant increase in the likelihood of oral and pharyngeal cancers [as a combined category] was reported for ever daily use of snus [hazard ratio of 3.1, 95% CI 1.5-6.6 based on 23 control and 11 cases for the combined diseases].
