# Supplementary material for: Snus: a compelling harm reduction alternative to cigarettes
Source: Harm Reduct J. 2019 Nov 27;16:62. doi: 10.1186/s12954-019-0335-1 (PMC6882181; doi:10.1186/s12954-019-0335-1)
Supplement: Supplementary file 7 — Additional file 7: Table S7. Epidemiological/clinical studies investigating the association between snus use and periodontal disease and/or gingival disease. Those epidemiological findings which are statistically significant (either protective or causative) are highlighted in red. N/A; not applicable. Klimisch Score adapted from Regulatory Toxicology and Pharmacology (1997) 25, 1-5 [118]. [file 12954_2019_335_MOESM7_ESM.docx]

| Study | Epidemiological/Clinical Findings | | | | |
| --- | --- | --- | --- | --- | --- |
| Modéer et al., 1980 [ref. 89] | **End-point** | **Number of subjects** | **Clinical Findings** | **95% Confidence Interval** | **Scoring assessment of quality of the study**  **(based on assessment using the Klimisch Score)** |
|  | Gingival Index | School children (n=232; boys, n=119; girls, n=113). Mean age 13.5 years. | Snus use reported by 11% of boys. No reported use in girls. Mean consumption of snus was five pinches per day with snus being present in the oral cavity for an average of 3.5 hours per day.  Only clinical endpoint which was statistically significant between snus users and non-users was gingival index on all surfaces as well as in the upper front jaw [after adjustment for plaque]. Gingival index in general was 1.10 for snus users and 0.89 in non-users. | N/A | 2 [small number of boys who self-reported as snus users (11% of 119)] |
| Frithiof et al., 1983 [ref. 95] | **End-point** | **Number of subjects** | **Clinical Findings** | **95% Confidence Interval** | **Scoring assessment of quality of the study**  **(based on assessment using the Klimisch Score)** |
|  | Gingival recession | Male snus users (n=21). Oral lesions were studied. | The lesions had a characteristic whitish appearance and the most common localization was in the vestibular area of the upper jaw. In two cases gingival retraction was observed. Light microscopical examination showed a mild epithelial dysplasia in five cases. No carcinoma in situ or invasive carcinoma was recognized. No evidence of gingival recession was observed | N/A | 2 [a small number of subjects were studied and no quantitative assessment of condition frequency was provided by the original authors] |
| Andersson and Axéll, 1989 [ref. 94] | End-point | **Number of cases** | **Clinical Findings** | **95% Confidence Interval** | **Scoring assessment of quality of the study**  **(based on assessment using the Klimisch Score)** |
|  | Gingival Recession | All subjects (n=252); Loose snus users, n=184; Portion-bag snuff users, n=68. | Clinical assessment conducted on all participants including clinical grading of lesions observed at sites where snus was placed. Gingival recession was also assessed. For a gingival recession to be registered as a snus-related recession, the borderline of the lesion should be in contact with the retracted gingival recession.  Among users of loose snus, 23.5% (n=42) subjects showed gingival recessions whilst only 2.9% (n=2) subjects who used portioned bagged snus showed gingival recessions. | N/A | 1 |
| Wickholm et al., 2004 [ref. 90] | **End-point** | **Number of cases** | **Odds Ratio (adjusted for age, gender, education, smoking and plaque)** | **95% Confidence Interval** | **Scoring assessment of quality of the study**  **(based on assessment using the Klimisch Score)** |
|  | Periodontal Disease  (defined as ≥3 teeth with pocket depth ≥5mm) | No snus use, n=1.521  Former snus use, n=31  Current snus use, n=122 | REFERENCE  2.55  0.66 | N/A  0.30-1.32  0.98-1.01 | 1 |
| Rolandsson et al., 2005 [ref.91] | **End-point** | **Number of subjects** | **Clinical Findings** | **95% Confidence Interval** | **Scoring assessment of quality of the study**  **(based on assessment using the Klimisch Score)** |
|  | Clinical assessment of snus associated lesions and general dental health including gingival recession | All subjects (n=80); Adolescent males aged 16 to 25 years. 40 snus users, 40 non-users. | Of the forty snus users, 35 showed snus induced lesions. The clinical diagnosis of snus users’ mucosa showed snus lesions of different severity clinically classified as degree 1, 2 and 3. When analysing snus lesions of degrees 2 and 3, hours of daily snus use and package form (portion-bag snus versus loose snus) was statistically significant. There were no statistical differences between snus users and non-users regarding restored tooth surfaces, presence of plaque, gingival inflammation and probing pocket depth. Seventeen percent of the cases showed loss of periodontal attachment as gingival recessions. | N/A | 1 |
| Bergström et al., 2006 [ref. 92] | **End-point** | **Number of subjects** | **Clinical Findings** | **95% Confidence Interval** | **Scoring assessment of quality of the study**  **(based on assessment using the Klimisch Score)** |
|  | Periodontal bone loss | All subjects (n=84; current snus users, n=25; former snus users, n=21; never users, n=38; aged 26-54) | Periodontal bone loss quantified as distance from cement-enamel junction (CEJ) to periodontal bone crest (PBC) at pre-molars and molars in each quadrant of the dentition. [stated as mean and 95% confident interval; Units are mm]  Current Snus Users 1.00, 0.87-1.13; Former Snus Users 1.12, 0.97-1.26; Never Users 1.06, 0.95-1.16. Association between snus use and bone height level controlling for age was not statistically significant (p>0.05). There was also no statistically significant difference between light and heavy exposure users controlling for age [light exposure defined as less than fifteen years of use; heavy use defined as more than fifteen years of use]. | N/A | 1 |
| Montén et al., 2006 [ref. 93] | **End-point** | **Number of cases/controls** | Odds Ratio (adjustment factors unknown) | **95% Confidence Interval** | **Scoring assessment of quality of the study**  **(based on assessment using the Klimisch Score)** |
|  | Gingival recession | Controls, n=70  Snus Users, n=33  All male; all non-smokers | ≥1 tooth with gingival recession (all teeth); **3.721**  ≥1 tooth with gingival recession (maxillary anterior tooth region); **5.099** | **1.401-9.886**  **1.672-15.549** | 1 |
| Hugoson et al., 2011 [ref. 96] | **End-point** | **Number of cases** | **Relative Risk** | **95% Confidence Interval** | **Scoring assessment of quality of the study**  **(based on assessment using the Klimisch Score)** |
|  | Periodontal disease | Cohort (n=1,591; 539 non-users of tobacco; 509 snus users; 543 conventional cigarette smokers) derived from three cohorts conducted ten years apart. | Participants were examined clinically and radiographically. Diagnostic criteria were number of teeth, plaque, gingival status, probing pocket depth ≥4mm, height of the alveolar bone level and classification by periodontal disease experience. Authors of the original study concluded that “*using snus did not seem to be a risk factor for periodontitis’.* | N/A | 1 |

**Supplementary Table 7**: Epidemiological/clinical studies investigating the association between snus use and periodontal disease and/or gingival disease. Those epidemiological findings which are statistically significant (either protective or causative) are highlighted in red. N/A; not applicable. Klimisch Score adapted from *Regulatory Toxicology and Pharmacology* (1997) **25**, 1-5 [118].
