# Supplementary material for: Snus: a compelling harm reduction alternative to cigarettes
Source: Harm Reduct J. 2019 Nov 27;16:62. doi: 10.1186/s12954-019-0335-1 (PMC6882181; doi:10.1186/s12954-019-0335-1)
Supplement: Supplementary file 8 — Additional file 8: Table S8. Epidemiological/clinical studies investigating the association between snus use and tooth loss and dental caries. Those epidemiological findings which are statistically significant (either protective or causative) are highlighted in red. N/A; not applicable. Klimisch Score adapted from Regulatory Toxicology and Pharmacology (1997) 25, 1-5 [118]. [file 12954_2019_335_MOESM8_ESM.docx]

| Study | Epidemiological/Clinical Findings | | | | |
| --- | --- | --- | --- | --- | --- |
| Hirsch et al., 1991 [ref. 97] | **End-point** | **Number of subjects** | **Clinical Findings** | **95% Confidence Interval** | **Scoring assessment of quality of the study**  **(based on assessment using the Klimisch Score)** |
|  | Dental Caries | Cohort (n=2,145; boys, n=1,122, girls, n=1,023; aged 14 to 19). 74% (n=1,574) self-reported as tobacco non-users, 17% (n=374) self-reported as conventional cigarette smokers, 9% (n=197) self-reported as snus users. | Three of the four clinical findings assessed (decayed missing and filled teeth, decayed filled proximal surface and initially delayed proximal surfaces) values were significantly higher in all groups for tobacco users [as an overall group] and for conventional cigarette smokers and snus users compared to those who used no form of tobacco. For decayed proximal surfaces [the fourth end-point assessed], values for significantly higher for tobacco users [as an overall group] only when compared to those who used no form of tobacco. | N/A | 2 [statistical assessment missing with regard to snus use for all groups other than for seventeen year -olds] |
| Rolandsson et al., 2005 [ref. 91] | **End-point** | **Number of subjects** | **Clinical Findings** | **95% Confidence Interval** | **Scoring assessment of quality of the study**  **(based on assessment using the Klimisch Score)** |
|  | Clinical assessment of snus associated lesions and general dental health including gingival recession | All subjects (n=80); Adolescent males aged 16 to 25 years. 40 snus users, 40 non-users. | Of the forty snus users, 35 showed snus induced lesions. The clinical diagnosis of snus users’ mucosa showed snus lesions of different severity clinically classified as degree 1, 2 and 3. When analysing snus lesions of degrees 2 and 3, hours of daily snus use and package form (portion-bag snus versus loose snus) was statistically significant. There were no statistical differences between snus users and non-users regarding restored tooth surfaces, presence of plaque, gingival inflammation and probing pocket depth. Seventeen percent of the cases showed loss of periodontal attachment as gingival recessions. | N/A | 1 |
| Hugoson et al., 2012 [ref. 98] | **End-point** | **Number of subjects** | **Clinical Findings** | **95% Confidence Interval** | **Scoring assessment of quality of the study**  **(based on assessment using the Klimisch Score)** |
|  | Dental Caries | Cohort (n=1,591; 1,142 non-users of tobacco; 104 snus users; 345 conventional cigarette smokers) derived from three cohorts conducted ten years apart. | Authors of the original study concluded that “*results of this cross-sectional study of three cohorts in 1983, 1993 and 2003 showed that daily smoking or snus does not increase the risk of dental caries in the studies carried out in 1993 and 2003*”. | N/A | 1 |
| Hellqvist et al., 2015 [ref.99]^1^ | **End-point** | **Number of subjects** | **Clinical Findings** | **95% Confidence Interval** | **Scoring assessment of quality of the study**  **(based on assessment using the Klimisch Score)** |
|  | Dental Caries | Snus Users (n=102; aged 26 to 62; habitual users >10 years). Control group (n=101; aged 29 to 61) | Initial Caries (snus users, 3.50±3.83; non-users, 3.00±3.94; p=0.264)  Manifest caries (snus users, 0.24±0.70; non-users, 0.33±0.78; p=0.406) | N/A | 1 |

**Supplementary Table 8**: Epidemiological/clinical studies investigating the association between snus use and tooth loss and dental caries. Those epidemiological findings which are statistically significant (either protective or causative) are highlighted in red. N/A; not applicable. Klimisch Score adapted from *Regulatory Toxicology and Pharmacology* (1997) **25**, 1-5 [118].

^1^This study does not provide epidemiological results in terms of quantification of effect of snus use on the risk of dental caries. Study provides quantitative measurement of several clinical end-points including enamel (initial) caries and dentine (manifest) caries. Dental caries quantified as the number of teeth based on a clinical visual assessment where “*loss of mineral in the enamel [is observed], giving a chalky appearance but without any clinical cavitations*” (initial caries) or “*new carious lesions on surfaces not previously restored and of such an extent that they could be verified as cavities by probing and, when probing fissures using light pressure, the probe became stuck*” (manifest caries) (p50 of the original article).
